# Targeting DDX3X Helicase Activity with BA103 Shows Promising Therapeutic Effects in Preclinical Glioblastoma Models

**DOI:** 10.3390/cancers13215569

**Published:** 2021-11-07

**Authors:** Annalaura Brai, Valentina Riva, Letizia Clementi, Lucia Falsitta, Claudio Zamperini, Virginia Sinigiani, Claudio Festuccia, Samantha Sabetta, Davide Aiello, Camilla Roselli, Anna Garbelli, Claudia Immacolata Trivisani, Laura Maccari, Francesca Bugli, Maurizio Sanguinetti, Pierpaolo Calandro, Mario Chiariello, Paola Quaranta, Lorenzo Botta, Adriano Angelucci, Giovanni Maga, Maurizio Botta

**Affiliations:** 1 Department of Biotechnology, Chemistry & Pharmacy, University of Siena, I-53100 Siena, Italy; annalaura.brai@unisi.it (A.B.); falsitta@student.unisi.it (L.F.); claudiozamperini@gmail.com (C.Z.); c.trivisani@gmail.com (C.I.T.); l.maccari@leaddiscoverysiena.it (L.M.); pierpa1986@gmail.com (P.C.); botta.maurizio@gmail.com (M.B.); 2 Istituto di Genetica Molecolare Luigi Luca Cavalli Sforza, IGM-CNR, Via Abbiategrasso 207, I-27100 Pavia, Italy; valentina.riva01@universitadipavia.it (V.R.); virginia.sinigiani01@universitadipavia.it (V.S.); elro.davide@gmail.com (D.A.); rosellic@tcd.ie (C.R.); agarbelli@gmail.com (A.G.); 3 Department of Biotechnological and Applied Clinical Sciences, University of L’Aquila, I-67100 L’Aquila, Italy; letizia.clementi@graduate.univaq.it (L.C.); claudio.festuccia@univaq.it (C.F.); samantha.sabetta@graduate.univaq.it (S.S.); 4 Dipartimento di Scienze Biotecnologiche di Base, Cliniche Intensivologiche e Perioperatorie, Università Cattolica del Sacro Cuore, 00168 Rome, Italy; fra.bugli@gmail.com (F.B.); maurizio.sanguinetti@unicatt.it (M.S.); 5 Dipartimento di Scienze di Laboratorio e Infettivologiche, Fondazione Policlinico Universitario A. Gemelli IRCCS, 00168 Rome, Italy; 6 Consiglio Nazionale delle Ricerche (CNR) and Core Research Laboratory (CRL), Istituto di Fisiologia Clinica (IFC), Istituto per lo Studio, la Prevenzione e la Rete Oncologica (ISPRO), Via Fiorentina 1, 53100 Siena, Italy; mario.chiariello@ittumori.it; 7 Department of Translational Research, University of Pisa, I-56127 Pisa, Italy; paola.quaranta@unipi.it; 8 Department of Ecological and Biological Sciences, University of Tuscia, Via S.C. De Lellis s.n.c., I-01100 Viterbo, Italy; lor.botta83@gmail.com; 9 Sbarro Institute for Cancer Research and Molecular Medicine, Center for Biotechnology, College of Science and Technology, BioLife Science Building, Suite 333, Temple University, 1900 N 12th Street, Philadelphia, PA 19122, USA

**Keywords:** DDX3X, glioblastoma, anticancer, xenograft, helicase inhibitors

## Abstract

**Simple Summary:**

In the last ten years, the human helicase protein DDX3X turned out to be an extremely interesting target for the development of potential anticancer drugs. Herein, we discovered BA103, a novel specific inhibitor of the helicase binding site of DDX3X, which is characterized by broad-spectrum anticancer activity. BA103 revealed promising tolerability in fibroblasts and good pharmacokinetic properties. Furthermore, BA103 was able to decrease the expression of β-catenin and to reduce tumor migration. Its capability to pass the blood–brain barrier led us to investigate its potential against glioblastoma, which is a high refractory disease with poor prognosis. High efficacy was proven in both xenograft and orthotopic animal models.

**Abstract:**

DDX3X is an ATP-dependent RNA helicase that has recently attracted interest for its involvement in viral replication and oncogenic progression. Starting from hit compounds previously identified by our group, we have designed and synthesized a new series of DDX3X inhibitors that effectively blocked its helicase activity. These new compounds were able to inhibit the proliferation of cell lines from different cancer types, also in DDX3X low-expressing cancer cell lines. According to the absorption, distribution, metabolism, elimination properties, and antitumoral activity, compound BA103 was chosen to be further investigated in glioblastoma models. BA103 determined a significant reduction in the proliferation and migration of U87 and U251 cells, downregulating the oncogenic protein β-catenin. An in vivo evaluation demonstrated that BA103 was able to reach the brain and reduce the tumor growth in xenograft and orthotopic models without evident side effects. This study represents the first demonstration that DDX3X-targeted small molecules are feasible and promising drugs also in glioblastoma.

## 1. Introduction

Cancer is a leading cause of death worldwide, accounting for 8.8 million deaths in 2018 [1]. The clinic-approved genotoxic/antimitotic chemotherapeutics, which interfere with basic biological processes, although potent, are characterized by undesired side effects that aggravate and undermine current therapeutic regimen. For these reasons, innovative targeted therapies acting against specific molecular targets are endowed with a critical role in tumor growth or progression: they represent a preferred strategy to improve the selectivity and safety profile of anticancer chemotherapy [2]. Successful examples of targeted cancer drugs are the monoclonal antibody Trastuzumab (Herceptin^®^), which is used to treat breast cancer overexpressing human epidermal growth factor receptor 2 protein (HER-2) [3], and Vemurafenib (Zelboraf^®^), a small molecule inhibitor of B-RAF protein, for the treatment of patients with inoperable or metastatic melanoma [4]. However, due to the genetic heterogeneity of solid tumors, which are usually endowed with alterations in several signaling pathways, it is often difficult to identify a single effective molecular target. For this reason, an alternative strategy could be represented by the design of inhibitors that are able to target multiple oncogenic pathways counteracting the intrinsic molecular resistance of cancer. In this context, the translation of oncogenes may well represent promising targets for anticancer therapy [5]. The DEAD-box RNA helicase family is involved in the modulation of transcription and translation of several genes, and it has been recently regarded as a useful target for selectively suppressing oncogene expression in several tumors [6].

DDX3X is a member of the DEAD-box ATP-dependent RNA helicase family, and it is ubiquitously expressed in human tissues. Its key roles in the modulation of innate immunity and viral replication prompted its early exploitation as an antiviral target. However, due to its involvement in different cellular pathways (translation, transcription, RNA decay, ribosome biogenesis) [7,8], it is being proposed as a novel therapeutic target also for the development of anticancer agents [9,10,11]. Interestingly, DDX3X biological importance has been linked to adaptive processes after cell stress. Several independent studies suggested that DDX3X participates in cell cycle progression, apoptosis, oncogenic transformation, migration, and hypoxia response; furthermore, it is overexpressed in a large number of cancers including prostate, lung, oral, ovarian, and hepatocellular carcinoma [9,10,11]. Experimental evidence showed that DDX3X in different tumors can act as an oncogene through the deregulation of the Wnt/β-catenin pathway or increasing Snail expression [12]. This aspect is particularly stimulating, because the Wnt/β-catenin pathway represents a consolidated and effective therapeutic target in many different cancer models. The knockdown of DDX3X reduced the basal expression of Snail, reducing cell proliferation and migration in HeLa and MCF-7 cells. Interestingly, a positive correlation between DDX3X levels and Snail expression was observed in 31 patients with glioblastoma multiforme (GBM) [13], which is an aggressive and malignant primary brain tumor that is characterized by poor survival rates. In some cases, DDX3X has been shown to act as an oncosuppressor by enhancing the p53-p21 axis; however, in tumors with non-functional p53, DDX3X has anti-apoptotic activity, promoting tumor growth by reducing caspase 3 activation [14]. Given that p53 is mutated in most tumor types and based on the oncogenic properties of DDX3X through the Wnt/β-catenin and Snail axes, targeting DDX3X could be an effective strategy for developing broad-spectrum anticancer therapies.

**Figure 1:**
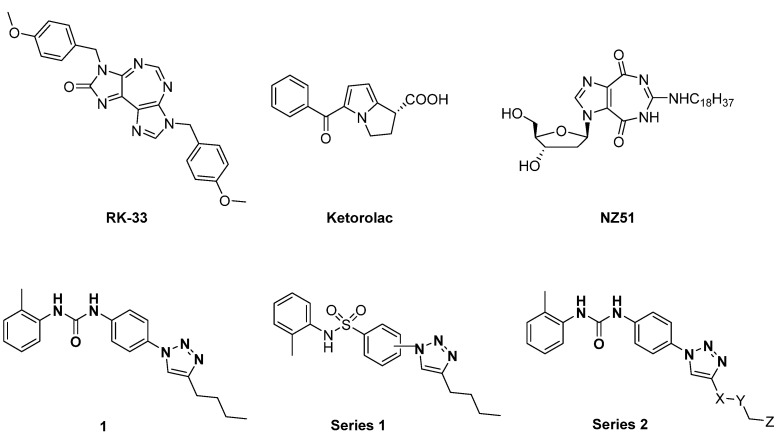
Two-dimensional (2D) structures of inhibitors of the DDX3X ATPase binging site published as anticancer compounds (upper line) and inhibitors of the DDX3X helicase binding site identified by replacing the urea of compound **1** with a sulfonamide (Series 1) and modifying the butyl side chain to increase the aqueous solubility (Series 2).

Recently, several small molecule inhibitors of DDX3X have been described as anti-cancer agents. Among the inhibitors of the DDX3X ATPase activity, compound RK-33 was able to reduce tumor volume in xenograft Ewing Sarcoma [15] and in preclinical lung and prostate cancer models in association with radiotherapy (Figure 1). Ketorolac salt decreased the number of neoplastic tongue lesions in a carcinogen-induced tongue tumor mouse model [16]. Finally, NZ51 was found effective against breast cancer in in vitro cellular assays [17]. These results build a solid basis for further investigations about the role of DDX3X inhibitors in targeting different tumors.

In the context of small molecules targeting DDX3X, our group previously identified the first ATPase inhibitors of this helicase, along with a large series of derivatives endowed with anti-HIV-1 activity [18]. Subsequently, in search of more selective DDX3X inhibitors, we identified for the first time a series of small molecules able to block the helicase activity of DDX3X [19,20], including compound **1** of this paper (Figure 1), which is endowed with broad-spectrum antiviral activity without any toxicity in preclinical models [19,20]. During the optimization studies, different families of DDX3X helicase inhibitors were identified, replacing the urea with a sulfonamide (Series 1) [21] and introducing polar substituents in the butyl side chain and small modification in the tolyl ring (Series 2, Figure 1) [20]. Based on these promising observations, in the present work, we developed novel DDX3X helicase inhibitors focusing our attention on the substitutions of triazole, tolyl, and central phenyl rings of the hit compound **1**, and after confirming the helicase inhibitory activity of the novel compounds, we screened them against a small panel of cancer cell lines, identifying potent inhibition growth (IG) on GBM cell lines. GBM is an aggressive brain tumor that is responsible for ≈200,000 deaths per year worldwide [22]. Despite multiple efforts in understanding GBM mechanism, the approved therapy is still represented by surgical resection of the tumor mass followed by radiotherapy or chemotherapy with the alkylating compound Temozolomide [23]. The challenges are represented by redundant pathways involved, resistance mechanisms, and inadequate delivery of drugs across the blood–brain barrier [24,25]. Even if immunotherapy and tumor-treating fields therapy have been approved in the last ten years, the prognosis remains poor, with survival rates lower than 5 years.

Due to the clinical importance of this difficult to treat brain tumor, compound **BA103** was selected for further in vivo studies, demonstrating good pharmacokinetic properties and potent activity in xenograft and orthotopic animal models. Even if additional studies should be performed to validate our compound using primary human GBM cells derived from patients, this study furnishes an in vivo proof of concept for the use of DDX3X inhibitors in GBM.

## 2. Materials and Methods

### 2.1. Chemistry

#### 2.1.1. General and Materials 

Reagents were obtained from commercial suppliers (for example Sigma-Aldrich, St. Louis, MO, USA; Alfa Aesar, Haverhill, MA, USA) and used as purchased without further purification. Anhydrous reactions were run under a positive pressure of dry N_2_. TLC was carried out using Merck (Kenilworth, NJ, USA) TLC plates silica gel 60 F254. Chromatographic purifications were performed using Merck 60 silica gel, 23–400 mesh.

#### 2.1.2. Instrumentation 

All NMR spectra were obtained on a Bruker Avance DPX400 spectrometer at 400 MHz for ^1^H-NMR or 100 MHz for ^13^C-NMR. The quantitative analysis was performed using an Agilent 1100 LC/MSD VL system (G1946C) (Agilent Technologies, Palo Alto, CA, USA) The LC-ESI-MS determination was performed by operating the MSD in the positive or negative ion mode. Spectra were acquired over the scan range 50–1500 m/z using a step size of 0.1 u. Chromatographic analysis was performed using a Varian Polaris 5 C18-A column (150 × 4.6 mm, 5 µm particle size) at rt. Analysis was carried out using a gradient elution of a binary solution; eluent A was ACN, while eluent B consisted of water. The analysis started at 0% A for three minutes; then, it rapidly increased up to 98% in 12 min and finally remained at 98% A until 18 min. The analysis was performed at a flow rate of 0.8 mL min^−1^, and the injection volume was 20 µL. The purity of compounds (as measured by peak area ratio) was >97%.

#### 2.1.3. Synthesis of Final Compounds

Full synthetic procedures are reported in Supporting Information.

1-(4-(5-Butylisoxazol-3-yl)phenyl)-3-(2-(trifluoromethyl)phenyl)urea (**2**): 4-(5-Butylisoxazol-3-yl)aniline (100 mg, 0.46 mmol) was added to a solution of the 1-(trifluoromethyl)phenyl isocyanate (85 µL, 0.65 mmol) in anhydrous CH_2_Cl_2_ (10 mL) in one portion. The solution was stirred for 4 h at room temperature under a nitrogen atmosphere. The solvent was removed at reduced pressure and the residue was purified by flash chromatography (PE/EtOAc 95:5) Yield 73%^1^HNMR (400 MHz, CDCl_3_): δ 8.00–7.98 (d, *J* = 7.6 Hz, 1H), 7.71–7.69 (d, *J* = 7.7 Hz, 2H), 7.58–7.48 (m, 2H), 7.42–7.40 (d, *J* = 7.7 Hz, 2H), 7.31–7.15 (m, 3H), 7.03 (s, 1H), 2.78–2.77 (t, *J* = 6.8 Hz, 2H), 1.71–1.69 (m, 2H), 1.44–1.40 (m, 2H), 0.96–0.93 (t, *J* = 6.8 Hz, 3H) ppm ^13^C-NMR (100 MHz, CDCl_3_): δ 170.27, 161.47, 153.67, 138.89, 137.42, 132.41, 127.75, 127.45, 127.20, 126.67, 126.32, 123.87, 123.81, 118.79, 106.46, 29.62, 29.20, 22.18, 14.01.ppm. HRMS (ESI) m/z calcd for C_21_H_20_F_3_N_3_O_2_ [M-H]^−^ 402.1429, found 402.1444. HPLC Purity: 97.2%.

1-(4-(5-Butyl-1,3,4-oxadiazol-2-yl)phenyl)-3-(2-(trifluoromethyl)phenyl)urea (**3**): Aniline **22** (31 mg, 0.14 mmol) was added to a solution of 2-(trifluoromethyl)phenyl isocyanate (22 µL, 0.15 mmol) in anhydrous CH_2_Cl_2_ (15 mL) in one portion. The solution was stirred for 9 h at r.t. under a nitrogen atmosphere. The solvent was removed at reduced pressure, and the residue was purified on silica to furnish the final product as a white solid. (Purification eluent: PE/EtOAc 7:3). Yield 71%, white solid. ^1^HNMR (400 MHz, MeOD-*d*_4_): δ 7.95–7.93 (m, 3H), 7.67–7.65 (m, 3H), 7.63–7.59 (t, *J* = 7. Hz, 1H), 7.31–7.27 (t, *J* = 7. Hz, 1H), 2.96–2.92 (t, *J* = 7.6, 2H), 1.86–1.79 (m, 2H), 1.51–1.42 (m, 2H), 1.01–0.97 (t, *J* = 7.2, 3H) ppm. ^13^C-NMR (100 MHz, ACETONE-*d*_6_): δ 166.35, 164.11, 152.03, 142.81, 136.48, 132.79, 127.35, 125.90, 125.55, 124.60, 123.65, 118.51, 29.30, 28.92, 21.82, 12.95 ppm. HRMS (ESI) m/z calcd for C_20_H_19_F_3_N_4_O_2_ [M-H]^−^ 403.1382, found 403.1401. HPLC Purity: 97.5%.

1-(4-(5-Butyl-4H-1,2,4-triazol-3-yl)phenyl)-3-(2-(trifluoromethyl)phenyl)urea (**4**): 4-(5-Butyl-4H-1,2,4-triazol-3-yl)aniline (25 mg, 0.11 mmol) was added to a solution of 2-(trifluoromethyl)phenyl isocyanate (18 µL, 0.11 mmol) in anhydrous CH_2_Cl_2_ (15 mL) in one portion. The solution was stirred for 12 h at r.t. under a nitrogen atmosphere. The solvent was removed at reduced pressure, and the residue was purified on silica to furnish the final product as a white solid. (Purification eluent: PE/EtOAc 7:3). Yield 60%, white solid. ^1^HNMR (400 MHz, MeOD-*d*_4_): δ 7.95–7.89 (m, 3H), 7.66–7.56 (m, 4H), 7.29–7.25 (t, *J* = 7.6 Hz, 1H), 2.81–2.77 (t, *J* = 7.6 Hz, 2H), 1.79–1.72 (m, 2H), 1.45–1.36 (m, 2H), 0.98–0.94 (t, *J* = 7.6 Hz, 3H) ppm.^13^C-NMR (100 MHz, MeOD-*d*_4_): δ 153.58, 146.64, 140.81, 135.86, 132.40, 126.77, 126.01, 125.67, 125.61, 124.01, 122.56, 121.78, 118.53, 29.31, 25.76, 21.88, 12.61 ppm. HRMS (ESI) m/z calcd for C_20_H_20_F_3_N_5_O [M-H]^−^ 403.1542, found 403.1580. HPLC Purity: 97.2%.

1-(4-(2-Butyl-2H-tetrazol-5-yl)phenyl)-3-(o-tolyl)urea (**5**). Compound **31** (0.10 mmol) was added to a solution of o-tolyl isocyanate (0.15 mmol) in anhydrous MeOH (10 mL) in one portion. The solution was stirred for 9 h at r.t. under a nitrogen atmosphere. The solvent was removed at reduced pressure and the residue was purified on silica to furnish the final product as a white solid. (DCM-MeOH 98:2). Yield 73% ^1^H NMR (Acetone-*d*_6_): δ 8.60 (s, 1H), 8.03–8.01 (d, *J* = 8.4 Hz, 2H), 7.92–7.90 (d, *J* = 8.4 Hz, 1H), 7.71–7.69 (d, *J* = 8.4 Hz, 2H), 7.55 (s, 1H), 7.18–7.14 (m, 2H), 6.99–6.95 (t, *J* = 7.6 Hz, 1H), 4.71–4.67 (t, *J =* 6.9 Hz, 2H), 2.27 (s, 3H), 2.03–1.98 (m, 2H), 1.43–1.34 (m, 2H), 0.97–0.94 (t, *J* = 7.4 Hz, 3H) ppm. ^13^C NMR (Acetone-d6): δ 164.91, 152.54, 142.16, 137.33, 130.35, 128.48, 127.64, 126.40, 123.44, 122.20, 121.37, 118.75, 52.47, 31.09, 19.34, 17.17, 12.73 ppm. HRMS (ESI) m/z calcd for C_19_H_22_N_6_O [M-H]^−^ 349.1777, found 349.1694. HPLC Purity: 99.6%.

1-(4-(2-Butyl-2H-tetrazol-5-yl)phenyl)-3-(2-(trifluoromethyl)phenyl)urea (**6**). Compound **31** (0.10 mmol) was added to a solution of o-trifluoromethyl-phenyl-isocyanate (0.15 mmol) in anhydrous MeOH (10 mL) in one portion. The solution was stirred for 9 h at r.t. under a nitrogen atmosphere. The solvent was removed at reduced pressure, and the residue was purified on silica to furnish the final product as white solid. (DCM-MeOH 98:2). Yield 70%, white solid. ^1^H NMR (400 MHz CDCl_3_): δ 8.28 (s, 1H), 7.93–7.91 (d, *J* = 8.4 Hz, 2H), 7.83–7.81 (d, *J* = 8.4 Hz, 1H), 7.47–7.34 (m, 3H), 7.09–7.05 (t, *J* = 7.2 Hz, 1H), 4.60–4.57 (t, *J* = 6.8 Hz, 2H), 2.01–1.97 (m, 2H), 1.39–1.33 (m, 2H), 0.95–0.91 (t, *J* = 7.2 Hz, 3H) ppm. ^13^C NMR (100 MHz, CDCl_3_): δ 164.70, 153.54, 140.20, 135.38, 132.54, 127.60, 126.29, 126.11, 125.23, 124.54, 122.43, 122.01, 120.07, 52.96, 31.27, 19.60, 13.34 ppm MS (ESI) m/z 405.1 [M + H]^+^, 428.1 [M + Na]^+^. HRMS (ESI) m/z calcd for C_19_H_19_F_3_N_6_O [M-H]^−^ 403.1494, found 403.1494. HPLC Purity: 99.5%.

1-(4-(2-(Ethoxymethyl)-2H-tetrazol-5-yl)phenyl)-3-(o-tolyl)urea (**7**). Compound **32** (0.10 mmol) was added to a solution of o-tolyl isocyanate (0.15 mmol) in anhydrous MeOH (10 mL) in one portion. The solution was stirred for 9 h at r.t. under a nitrogen atmosphere. The solvent was removed at reduced pressure, and the residue was purified on silica to furnish the final product as a white solid. (DCM-MeOH 98:2). Yield 62% ^1^H NMR (400 MHz CDCl_3_): δ 8.06–8.04 (d, *J* = 8.0 Hz, 2H), 7.65–7.61 (m, 3H), 7.21–7.15 (m, 2H), 7.05–7.01 (t, *J* = 7.6 Hz, 1H), 5.96 (s, 2H), 3.75–3.70 (q, *J* = 6.8 Hz, 2H), 2.30 (s, 3H), 1.21–1.17 (t, 3H, *J* = 6.8 Hz) ppm. ^13^C NMR (100 MHz CDCl_3_): δ 165.24, 142.06, 136.14, 130.47, 127.51, 126.33, 124.73, 123.05, 120.95, 118.61, 80.93, 66.18, 16.61, 13.32 ppm.

HRMS (ESI) m/z calcd for C_18_H_20_N_6_O_2_ [M-H]^−^ 351.1569, found 351.1591. HPLC Purity: 98.1%.

1-(4-(5-Butyl-1,3,4-thiadiazol-2-yl)phenyl)-3-(2-(trifluoromethyl)phenyl)urea (**8**): 4-(5-Butyl-1,3,4-thiadiazol-2-yl)aniline (25 mg, 0.11 mmol) was added to a solution of 2-(trifluoromethyl)phenyl isocyanate (17 µL, 0.11 mmol) in anhydrous CH_2_Cl_2_ (15 mL) in one portion. The solution was stirred for 12 h at r.t. under a nitrogen atmosphere. The solvent was removed at reduced pressure, and the residue was purified on silica to furnish the final product as a white solid. (Purification eluent: PE/EtOAc 7:3). Yield 68%, white solid. ^1^H NMR (400 MHz, CDCl_3_) δ 8.93 (s, 1H), 7.89–7.85 (m, 2H), 7.74–7.72 (d, *J* = 7.9 Hz, 2H), 7.55–7.53 (d, *J* = 7.2 Hz, 2H), 7.48–7.46 (d, *J* = 8.4 Hz, 2H), 7.17–7.14 (t, *J* = 7.5, 1H), 3.10–3.07 (t, *J* = 7.4 Hz, 2H), 1.78–1.75 (m, 2H), 1.45–1.39 (m, 2H), 0.93–0.89 (t, *J* = 7.4 Hz, 3H) ppm. ^13^C NMR (100 MHz CDCl_3_):170.62, 168.98, 153.37, 141.94, 135.57, 132.53, 128.66, 126.62, 126.10, 124.56, 124.14, 122.52, 119.50, 32.07, 29.84, 22.11, 13.60 ppm. HRMS (ESI) m/z calcd for C_20_H_19_F_3_N_4_OS [M-H]^−^ 419.11, found 419.1121. HPLC Purity: 97.9%.

1-(6-(4-Isopentyl-1H-1,2,3-triazol-1-yl)pyridin-3-yl)-3-(2-(trifluoromethyl) phenyl)urea (**9**). Aniline **31** (0.10 mmol) was added to a solution of 2-(trifluoromethyl)phenyl isocyanate (0.15 mmol) in anhydrous CH_2_Cl_2_ (15 mL) in one portion. The solution was stirred for 9 h at r.t. under a nitrogen atmosphere. The solvent was removed at reduced pressure, and the residue was purified on silica to furnish the final product as white solid. (Purification eluent: DCM-MeOH 98:2). Yield 61%, white solid. ^1^H NMR (400 MHz, Acetone-*d*_6_): δ 9.14 (s, 1H), 8.66 (s, 1H), 8.37 (s, 1H), 8.28–8.24 (d, *J* = 8.0 Hz, 1H), 8.14–8.12 (d, *J* = 8.0 Hz, 1H), 8.06–8.04 (d, *J =* 8.0 Hz, 1H), 7.81 (s, 1H), 7.70–7.64 (m, 2H), 7.33–7.30 (t, *J =* 8.0 Hz, 1H), 2.80–2.75 (t, *J =* 7.7 Hz, 2H), 1.64–1.61 (m, 3H), 0.96–0.94 (d, *J =* 8.0 Hz, 6H) ppm. ^13^C NMR (100 MHz, Acetone-*d*_6_): δ 152.31, 148.41, 144.17, 138.61, 138.26, 136.46, 132.84, 128.80, 125.95, 125.65, 123.96, 117.98, 117.71, 113.35, 38.34, 27.33, 23.23, 21.91, 21.64 ppm. HRMS (ESI) m/z calcd for C_20_H_21_N_6_O [M-H]^−^ 417.1615, found 417.1679. HPLC Purity: 99.3%.

1-(6-(4-Isopentyl-1H-1,2,3-triazol-1-yl)pyridin-3-yl)-3-(isoquinolin-5-yl)urea (**10**): (Purification eluent: DCM-MeOH 95:5). Yield 59%, white solid. ^1^H NMR (100 MHz, CDCl_3_) δ = 9.05 (s, 1H), 8.98 (s, 1H), 8.31–8.30 (d, *J* = 5.6 Hz, 1H), 8.15–8.12 (m, 2H), 8.06–8.03 (d, *J* = 12.0 Hz, 1H), 7.88–7.86 (d, *J* = 8.8 Hz, 1H), 7.72–7.71 (d, *J* = 4.4 Hz, 1H), 7.63–7.61 (d, *J* = 8.0 Hz, 1H), 7.52–7.49 (t, *J* = 7.8 Hz, 1H), 2.71–2.67 (t, *J* = 7.4 Hz, 2H), 1.54–1.51 (m, 3H), 0.86–0.84 (d, *J* = 6.0 Hz, 3H) ppm. ^13^C NMR (100 MHz, CDCl_3_) δ = 153.29, 152.43, 148.88, 143.67, 141.83, 138.35, 136.24, 132.67, 129.51, 129.01, 128.65, 127.75, 123.69, 123.14, 118.21, 114.56, 113.80, 38.18, 27.47, 23.34, 22.19. HRMS (ESI) m/z calcd for C_22_H_23_N_7_O [M-H]^−^ 400.1886, found 400.1855. HPLC Purity: 99.5%.

#### 2.1.4. General Procedure for the Preparation of Compounds 11–14 

The opportune aniline **46** or **47** (100 mg, 0.46 mmol) was added to a solution of the appropriate isocyanate (85 µL, 0.65 mmol) in anhydrous CH_2_Cl_2_ (10 mL) in one portion. The solution was stirred for 4 h at r.t. under a nitrogen atmosphere. The solvent was removed at reduced pressure, and the residue was purified by flash chromatography using the opportune eluent.

1-(4-(4-Butyl-1H-1,2,3-triazol-1-yl)phenyl)-3-(2-(trifluoromethyl)phenyl)urea (**11**). (Purification eluent: DCM/MeOH 98:2). Yield 78%, white solid. ^1^HNMR (400 MHz, MeOD-*d*_4_): δ 8.16 (s, 1H), 7.93–7.92 (d, *J =* 8.0 Hz, 2H), 7.71–7.68 (m, 2H), 7.64–7.61 (m, 3H), 7.59–7.55 (t, *J =* 7.8 Hz, 1H), 7.27–7.23 (t, *J=* 8.0 Hz, 1H), 2.75–2.71 (t, *J =* 7.6 Hz, 2H), 1.72–1.64 (m, 2H), 1.42–1.34 (m, 2H), 0.96–0.92 (t, *J=* 7.6 Hz, 3H) ppm. ^13^C NMR (100 MHz, MeOD-*d*_4_): δ 153.59, 148.64, 139.83, 136.14, 132.43, 131.98, 126.07, 125.71, 124.12, 120.79, 119.82, 119.33, 31.28, 24.61, 21.88, 12.71 ppm. HRMS (ESI) m/z calcd for C_20_H_20_F_3_N_5_O [M-H]^−^ 402.1542, found 402.1599. HPLC Purity: 99.6%.

1-(1-Chloro-3-methylisoquinolin-4-yl)-3-(4-(4-isopentyl-1H-1,2,3-triazol-1-yl)phenyl)urea (**12**): (Purification eluent: DCM/MeOH 98:2). Yield 60%, white solid. ^1^HNMR (400 MHz, MeOD-*d*_4_): δ 8.34–8.32 (d, *J* = 8.4 Hz, 1H), 8.21 (s, 1H), 8.08–8.06 (d, *J* = 8.4 Hz, 1H), 7.89–7.85 (t, *J* = 7.6 Hz, 1H), 7.74, 7.20 (m, 3H), 7.67–7.65 (d, *J* = 8.4 Hz, 2H), 2.79–2.75 (t, *J=* 7.2 Hz, 2H), 2.62 (s, 3H), 1.64–1.61 (m, 3H), 0.93–0.92 (d, *J* = 6.0 Hz, 6H) ppm. ^13^CNMR (100 MHz CDCl_3_): δ = 153.28, 153.18, 151.88, 147.67, 136.75, 135.75, 133.97, 129.85, 128.14, 127.77, 127.55, 125.89, 123.06, 123.03, 121.02, 119.62, 38.27, 27.88, 27.45, 22.84, 21.44 ppm. HRMS (ESI) m/z calcd for C_24_H_25_ClN_6_O [M-H]^−^ 447.1700, found 447.1658. HPLC Purity: 97.2%.

1-(4-(4-Isopentyl-1H-1,2,3-triazol-1-yl)phenyl)-3-(4-methylpyridin-3-yl)urea (**13**). The residue was purified by flash chromatography on silica gel (DCM/MeOH 98:2). Yield 74%, white solid. ^1^H NMR (400 MHz, MeOD-*d*_4_): δ 8.67 (s, 1H), 8.20 (s, 1H), 8.15–8.14 (d, *J* = 4.8 Hz, 1H), 7.84–7.81 (d, *J* = 8.0 Hz, 2H), 7.67–7.65 (*J* = 8.0 Hz, 2H), 7.30–7.29 (d, *J* = 4.8 Hz, 1H), 2.78–2.74 (t, *J* = 8.0 Hz, 2H), 2.35 (s, 3H), 1.63–1.60 (m, 3H), 0.97–0.95 (d, *J* = 8.0 Hz, 6H) ppm. ^13^C-NMR (100 MHz, MeOD-*d*_4_): δ 164.69, 153.67, 143.82, 143.19, 141.39, 140.12, 134.50, 127.09, 125.50, 121.37, 118.72, 38.28, 27.38, 22.87, 21.32, 16.59 ppm. HRMS (ESI) m/z calcd for C_19_H_22_N_6_O [M-H]^−^ 349.1777, found 349.1777. HPLC Purity: 98.3%.

1-(4-(4-Butyl-1H-1,2,3-triazol-1-yl)phenyl)-3-(4-methylpyridin-3-yl)urea (**14**). The residue was purified by flash chromatography on silica gel (DCM/MeOH 98:2). Yield 68%, white solid. ^1^H NMR (400 MHz, MeOD-*d*_4_): δ 8.87 (s, 1H), 8.20 (s, 1H), 8.15–8.14 (d, *J* = 4 Hz, 1H), 7.74–7.71 (d, *J* = 8.0 Hz, 2H), 7.65–7.63 (*J* = 8.0 Hz, 2H), 7.30–7.29 (d, *J* = 4 Hz, 1H), 4.70–4.67 (t, *J* = 7.0 Hz, 2H), 2.36 (s, 3H), 2.06–1.99 (m, 2H), 1.41–1.36 (m, 2H), 1.00–0.96 (t, *J* = 8.0 Hz, 3H) ppm. ^13^C-NMR (100 MHz, MeOD-*d*_4_): δ164.69, 153.67, 143.82, 143.19, 141.39, 140.12, 134.50, 127.09, 125.50, 121.37, 118.72, 50.61, 30.06, 18.51, 14.20, 11.30 ppm. HRMS (ESI) m/z calcd for C_20_H_24_N_6_O [M-H]^−^ 363.1933, found 363.1976. HPLC Purity: 98.1%.

### 2.2. In Silico Studies

#### 2.2.1. Docking Studies

All compounds studied herein were docked within the RNA binding site of the modeled hDDX3X closed conformation using the software package GOLD 5.2. The pocket under investigation was inserted into a grid box centered on residue Arg276 and enclosing residues lying within 10 Å from such amino acid. The genetic algorithm parameter settings were employed using the search efficiency set at 100%; we increased the number of GA runs from 10 to 30. We performed a consensus scoring using PLP as the fitness function, and we rescored all poses with ChemScore software. For each inhibitor, the first ranked solution was selected for further analysis.

#### 2.2.2. Molecular Dynamic Methods

The homology model was used to execute a Molecular Dynamic (MD) simulation using Amber 16. This model represents the closed conformation of human DDX3X, in complex with ATP and Mg^2+^. The parametrization of compounds was performed using the antechamber module available in Amber 16. Atom types were assigned using GAFF (Generalized Amber Force Field), while charges were assigned with the AM1-BCC method. Protein was described using the ff14SB force field. The system was solvated using a 15Å octahedral box of TIP3P water molecules, and the complex was neutralized by adding the appropriate number of Na+ ions. Four steps of minimization were executed to remove bad contacts. Three of these steps were provided for the minimization of water molecules and side chains of the protein and ligand molecule. The last minimization, conducted on the whole system, consisted of 10,000 steps divided into 1000 steps of steepest descent and 9000 steps of conjugate gradient. In the first step of MD calculation, the temperature was increased from 0 to 300 K with constant volume using the Langevin method, while the second step was performed using Berendsen barostat to control pressure. After the system equilibration, 100 ns of MD simulation were run using the SHAKE algorithm to treat hydrogen-containing bonds. Trajectories were analyzed using VMD.

### 2.3. Enzymatic Assays

#### 2.3.1. Protein Expression and Purification

Recombinant his-tagged human full length DDX3X cloned the *E. coli* expression vector pET-30a(+). ShuffleT7 *E. coli* cells were transformed with the plasmid and grown at 37 °C up to OD_600_ = 0.7. DDX3X expression was induced with 0.5 mM IPTG at 15 °C O/N. Cells were harvested by centrifugation, lysed, and the crude extract was centrifuged at 100,000 g for 60 min at 4 °C in a Beckman centrifuge before being loaded onto a FPLC Ni-NTA column (GE Healthcare, Chicago, IL, USA). The column was equilibrated in Buffer A (50 mM Tris-HCl pH 8.0, 250 mM NaCl, 25 mM Imidazole and 20% glycerol). After extensive washing in Buffer A, the column was eluted with a linear gradient in Buffer A from 25 to 250 mM Imidazole. Proteins in the eluted fractions were visualized on SDS-PAGE and tested for the presence of DDX3X by Western blot with anti-DDX3X A300-475A (Bethyl Laboratories, Montgomery, TX, USA) at 1:4000 dilution in 5% milk. Fractions containing the purest DDX3X protein were pooled and stored at −80 °C.

#### 2.3.2. Helicase Assay Based on Fluorescence Resonance Energy Transfer (FRET)

The dsRNA substrate for the helicase assay was prepared by hybridizing two ss RNA oligonucleotides with the following sequences:

Fluo-FAM 5′ UUUUUUUUUUUUUUAGUACCGCCACCCUCAGAACC 3′
Qu-BHQ1 5′ GGUUCUGAGGGUGGCGGUACUA 3′
DNA capture 5′ TAGTACCGCCACCCTCAGAACC 3′.

The sequence in Fluo-FAM complementary to Qu-BHQ1 is underlined. Fluo-FAM carries a 6-carboxyfluorescein fluorophore at its 3′ end, while Qu-BHQ1 carries a Black Hole quencher group at its 5′ end. The DNA capture oligonucleotide is complementary to the Qu-BHQ1 oligonucleotide, but it bears no modifications. A helicase assay using the dsRNA substrate was performed in 20 mM TrisHCl (pH 8), 70 mM KCl, 2 mM MgCl_2_, 2 mM dithiothreititol, 12 units RNasin (Promega), 2 mM ATP, 50 nM dsRNA, and 100 nM capture strand in 20 μL of reaction volume. The unwinding reaction was started by adding 60 pmols of DDX3X recombinant protein and carried out at 37 °C for 40 min using a LightCycler 480 (Roche, Basel, Switzerland). The fluorescence intensity was recorded every 30 s. Values of fluorescence signal in the presence or in the absence of inhibitor molecules were analyzed by linear interpolation, and the corresponding slope values were used to determine the apparent unwinding rate.

#### 2.3.3. Kinetic Analysis

The IC_50_ values have been calculated from dose–response curves. Data (in triplicate) were plotted and analyzed by least-squares nonlinear regression, according to the method of Marquardt–Levenberg, with the computer program GraphPad Prism 6.0. Data were fitted to the following equation:

E_obs_ = E_max_/(1 + (IC_50_/[I])^n^) (1)

where E_(obs)_ is the observed enzymatic activity in the presence of each inhibitor dose [I]; E_(max)_ is the maximal enzymatic activity in the absence of the inhibitor; and n is an exponential term to take into account sigmoidal dose–response curves.

#### 2.3.4. Cell Extracts and DDX3X Quantification

The procedure for DDX3X quantification was previously published in [26]. Briefly, 10^7^ cells were ruptured with a Dounce homogenizer in Lysis Buffer (50 mM Tris HCl pH 8.0, 0.1% SDS, 350 mM NaCl, 0.25% Triton X-100, protease inhibitor cocktail (Sigma-Aldrich). The lysate was incubated for 30 min on ice, sonicated for 5 min (at 30 s intervals), and centrifuged at 20,000× *g* for 10 min at 4 °C. The protein concentration in the supernatant (crude extract) was quantified with Bradford. For DDX3X quantification, increasing concentrations of crude extract (5 µg, 10 µg, 20 µg, 40 µg) were loaded on SDS-PAGE alongside known concentrations (50 ng, 100 ng, 150 ng, 200 ng) of recombinant purified DDX3X. Separated proteins were subjected to Western blot analysis with anti-DDX3X A300-475A (Bethyl Laboratories, Montgomery, TX, USA) at 1:4000 dilution in 5% milk. The blot was next incubated with a HRP-conjugated secondary antibody, and the bands corresponding to DDX3X were visualized with Enhanced Chemiluminescent Substrate (Westar Nova 2.0, Cyanagen, Bologna, Italy) using a ChemiDoc™ XRS (Bio-Rad, Hercules, CA, USA) apparatus. The intensity of the bands was measured by densitometry, and the values obtained for the purified DDX3X were plotted as a function of the protein concentration and analyzed by linear interpolation to derive a reference curve, whose slope corresponded to the estimated intensity (I) × ng^−1^ (DDX3X) value. This parameter was used to calculate the I × ng^−1^ values for the DDX3X in the cell extract from the intensities of the DDX3X bands in the corresponding cell extract (CE) samples. The mean I × ng^−1^ (CE) value was used to calculate the ng of DDX3X × µl^−1^ of extract and then the total ng of DDX3X present in the extract. This value was divided for the total number of cells used (10^7^) to derive the ng DDX3X/cell. Based on the known molecular weight of DDX3X, the Avogadro number, and assuming a mean cellular volume of 6.55 × 10^−11^ L, the final molar concentration of DDX3X per cell was calculated. Each experiment was repeated three times, with each blot carrying a reference curve alongside the extract samples, to account for variations in loading/transfer efficiency. The reference I × ng^−1^ (DDX3X) value obtained was comparable across the different experiments (±20% variation).

### 2.4. Cellular Assay

#### 2.4.1. Cell Cultures and Reagents

Cells were purchased from American Type Culture Collection (ATCC, Manassas, VA, USA). The LNCaP (ATCC^®^ CRL-1740™), A549 (ATCC^®^ CCL-185™), MDA-MB-231 (ATCC^®^ CRM-HTB-26™), 22Rv1 (ATCC^®^ CRL-2505™), HCT-116 (ATCC^®^ CCL-247™), and PC3 (ATCC^®^ CRL-1435™) cells were maintained in Roswell Park Memorial Institute 1640 medium (RPMI; Euroclone, ECB9006L) supplemented with 10% fetal bovine serum (Euroclone; ECS0180L), 2 mM L-glutamine, 100 units/mL penicillin, and 100 mg/mL streptomycin at 37 °C in an atmosphere of 5% CO_2_/air. DU145 (ATCC^®^ HTB-81™) and DAOY (ATCC^®^ HTB-186™) cells were maintained in Eagle’s minimal essential medium (EMEM; Euro-clone, ECM0445L) supplemented with 10% fetal bovine serum (Euroclone; ECS0180L), 2 mM L-glutamine, 100 units/mL penicillin, and 100 mg/mL streptomycin at 37 °C in an atmosphere of 5% CO_2_/ air. SH-SY5Y (ATCC^®^ CRL-2266™), DBRTG (ATCC^®^ CRL-2020™), HeLa (ATCC^®^ CCL-2™), HN6 cells (kindly provided by Dr Silvio Gutkind, UCSD Medical Center, Moores Cancer Center USA, Chen J.J et al., Oncotarget2013, 4, 206–217), U2OS (ATCC^®^ HTB-96™), U87 (ATCC^®^ HTB-914™), U251 (ATCC^®^ HTB-14™), and RD18 cells (kindly provided by Dr P. Boccuni Memorial Sloan-Kettering Cancer Center, New York, USA Vella S. et al. ClinEpigenetics. 2015 6; 7–82) were maintained in Dulbecco’s modified Eagle’s medium (DMEM; Euroclone, ECB7501L) supplemented with 10% fetal bovine serum (Euroclone; ECS0180L), 2 mM L-glutamine, 100 units/mL penicillin, and 100 mg/mL streptomycin at 37 °C in an atmosphere of 5% CO_2_/air.

#### 2.4.2. Cytotoxicity Assay 

For CellTiter 96^®^ Aqueous One Solution Cell Proliferation Assay (MTS) (Promega, Madison, WI, USA), cells were seeded at 5 × 10^3^ per well in 96-well plates in quadruplicate together a control with no cells to evaluate the background. After 24 h, the culture medium was removed, and fresh medium containing increasing concentrations of chemical compounds was added to cells. The fixed final concentrations were 0.1 μM, 1 μM, 10 μM, and 100 μM. DMSO was the vehicle used for dilution of the compounds, and its final concentration on cells was less than 0.2%. Medium-only containing wells were included as controls. Cells were incubated with compounds at different concentrations for 72 h, then, 20 µL of CellTiter 96^®^ AQueous One Solution Reagent was added into each well. After 3 h at 37 °C in a humidified, 5% CO_2_ atmosphere, the absorbance at 492 nm was recorded using an EZREAD400 (Biochrom, Cambridge, United Kingdom) plate reader. The average 490 nm absorbance from the “no cell” control wells was subtracted from all other values to yield background-corrected absorbance. Dose–response curves and CC_50_ values have been calculated by plotting the absorbance data as a function of inhibitor concentrations and fitting the data by least-squares nonlinear regression, according to the method of Marquardt–Levenberg, with the computer program GraphPad Prism 6.0.

#### 2.4.3. Cell Cycle Analysis

Exponential growing cells were treated with selected inhibitors at the density of 1 × 10^6^ cells/mL for 48 h. At the end point, at least 5 × 10^6^ cells were harvested, washed with phosphate buffer saline (PBS), and fixed overnight with 70% ethanol. Then, ethanol was removed by centrifugation, and the cells were resuspended in PBS and stained with 50 μg/mL propidium iodide (PI) at 4 °C for 30 min in the dark. Stained cells were analyzed by a Tali image-based cytometer (Life Technologies, Carlsbad, CA, USA) counting 20 fields per sample, and exported fcs raw data were elaborated by Flowing software (v. 2.5.0, by Perttu Terho, University of Turku, Finland).

#### 2.4.4. Migration Assay

Exponential growing cells were treated with the inhibitor or vehicle for 48 h and then trypsinized, washed twice with PBS, rinsed in complete medium, incubated at 37 °C for 30 min to reconstitute the membrane structures, washed again for FCS removal, and then added to the upper compartment of collagen-coated (PTFE) Transwell insert (Corning Inc., Corning, NY, USA). Cells were allowed to migrate through coated filters for 6 h. The cells attached on the lower membrane surfaces were stained with 0.1% crystal violet w/v in 0.1 mol/L borate, pH 9.0, and 2% ethanol *v*/*v* for 20 min at room temperature. Cells were counted at 400× magnification in standard optical microscopy, and the average number of cells per field in 5 random fields was recorded. Triplicate filters were used, and the experiments were repeated at least two times.

#### 2.4.5. Western Blot

Cells were incubated for 15 min in lysis buffer containing 1% triton, 0.1% SDS, 2 mmol/L CaCl2, 10 mg/mL orthovanadate, and 1× protease inhibitors cocktail (Sigma-Aldrich, St. Louis, MO, USA). Forty micrograms of proteins were electrophoresed in 12% SDS-polyacrylamide gel, and then, the gel was placed onto a Trans-Blot Turbo mini nitrocellulose transfer pack and transferred using a Trans-Blot Turbo Transfer System (Bio-Rad Laboratories, Hercules, CA, USA). The membrane was incubated with 1 mg/mL primary antibody and then with 0.5 mg/mL horseradish peroxidase (HRP)-conjugated secondary antibodies (Santa Cruz Biotechnology, Dallas, TX, USA). Primary antibody against DDX3X was purchased from Bethyl laboratories Inc. (#A300-474A), β-catenin (H102), and GAPDH from Santa Cruz Biotechnology. Protein bands were visualized using a chemiluminescent detection system (Thermo Scientific, Waltham, MA, USA), and signals were digitally acquired by Chemidoc XRS system (Bio-Rad Laboratories). Analysis of band MW and density was performed with Image Lab software (Bio-Rad Laboratories).

#### 2.4.6. Immunofluorescence Assay

Cells grown on coverslips (2 × 10^4^ cells/cm^2^) were fixed in 4% w/v paraformaldehyde in PBS for 10 min at RT and permeabilized in PBS containing 0.1% *v*/*v* Triton X-100 for 5 min at RT. Then, cells were incubated with 10 μg/mL primary antibody (β-catenin, H-102 Santa Cruz Biotechnology) and diluted in PBS containing 3% w/v bovine serum albumin (BSA) for 1 h at RT. After three washes with PBS, cells were treated with fluorescein-labeled IgG secondary antibody (1:100 in PBS containing 3% w/v BSA) for 30 min at RT. After extensive washings, cells were mounted with ProLong Gold antifade mounting medium (Life Technologies Corporation, Carlsbad, CA, USA) and observed by a fluorescence microscope equipped with a digital camera (AXIOPHOT, Carl Zeiss, Oberkochen, Germany).

### 2.5. In Vivo Studies

#### 2.5.1. Subcutaneous Xenograft

Male CD1 nude mice (Charles River, Milan, Italy) were maintained under the guidelines established by our Institution (University of L’Aquila), complying with the Declaration of Helsinki, national and international laws and policies (European Economic Community Council Directive 86/609; Italian Legislative Decree 4.03.2014 n.26, Gazzetta Ufficiale della Repubblica Italiana no. 61, 4 March 2014). Before any invasive manipulation, mice were anesthetized with a mixture of ketamine (25 mg/mL)/xylazine (5 mg/mL). Tumor grafts were obtained by injecting s.c. 1 × 10^6^ U87 cells in 100 µL of 12 mg/mL Matrigel (Becton Dickinson, Franklin Lakes, NJ, USA). Tumor growth was monitored daily by measuring the average tumor diameter, and mice were divided into experimental groups when the tumor reached a volume of approximately 50 mm^3^. The tumor volume was calculated according to the formula 4/3πr^3^, with r representing the mean diameter. BA103 was administered i.p. in a saline solution containing 10% *v*/*v* DMSO and 10% *v*/*v* TWEEN 80. Each mouse received 50 mg/kg BA103, or vehicle, three times per week. Previous experiments with the same settings have permitted choosing the endpoint of 21 days considering the maximum mean volume of tumors in control mice that was compatible with no signs of distress [27]. At the end point, tumor masses were excised and weighed.

#### 2.5.2. Immunohistochemistry

Xenograft tumor mass was fixed in 4% w/v paraformaldehyde for 24 h and then included in paraffin. Slide-mounted tissue sections (4 um thick) were deparaffinized in xylene and hydrated serially in 100%, 95%, and 80% ethanol. For immunohistochemistry, endogenous peroxidases were quenched in 3% H_2_O_2_ in PBS for 1 h, and then, slides were incubated with a primary antibody for 1 h at room temperature (DDX3X, Bethyl laboratories Inc.). Sections were washed three times in PBS, and antibody binding was revealed using the Sigma fast 3,30-diaminobenzidine tablet set (Sigma). The sections were washed, dehydrated, and mounted with resinous mounting medium.

#### 2.5.3. Orthotopic Xenograft 

In CD1 nude mice, 3 × 10^3^ U87 cells were injecting intracerebrally using a stereotactic frame (Stoelting Europe, Dublin, Ireland). Optimization of the protocol for treatment procedures was based on previously published data performed in the same experimental in vivo model [27]. A volume of 3 μL was inoculated by a sterile Hamilton syringe with a 26-gauge needle inserted at a depth of 3.0 mm from the skull surface. Five days after injection, animals were randomized to control and treated groups. Mice were euthanized when they displayed neurological signs (e.g., altered gait, tremors/seizures, lethargy) or weight loss of 20% or greater respect to control group. For in vivo administration, BA103 was administered i.p. in a saline solution containing 10% *v*/*v* DMSO and 10% *v*/*v* TWEEN 80. Each mouse received 50 mg/kg BA103, or vehicle, three times per week.

### 2.6. Data Analysis and Statistics

Data are expressed as means ± standard deviations (SDs) of at least three independent experiments. All the statistical procedures were performed by Graph-Pad Prism Software Inc. (San Diego, CA, USA). The statistical significance between measure series was calculated with a parametric Student’s *t*-test and *p* values of less than 0.01.

## 3. Results

### 3.1. Design and Synthesis of Compounds

Novel derivatives of compound **1** were rationally designed based on the previously reported SAR using a homology model of human DDX3X in complex with ATP and Mg^2+^ (closed conformation). The model was generated employing Prime [28,29] (PDB structures 2DB3 and 4PXA). The docking score and adsorption distribution, metabolism, and excretion (ADME) properties were taken into account to design the novel compounds using QikProp software [30].

**Figure 2:**
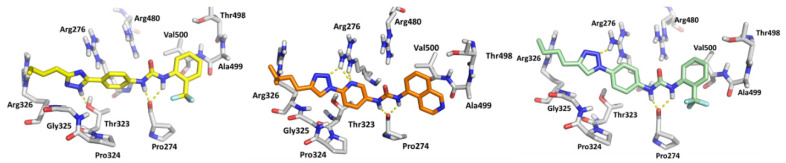
Predicted binding mode of compounds 4 (yellow), 10 (orange), and BA103 (light green) in the RNA-binding site. Hydrogen bond interactions are represented in yellow lines (Supplementary Information for other selected compounds in Figure S1).

All the compounds studied herein were docked within the RNA-binding site of the modeled DDX3X in closed conformation using the software package GOLD 5.2. The pocket under investigation was inserted into a grid box centered on residue Arg276 and enclosing residues lying within 10 Å from such an amino acid. The predicted binding mode of compounds 4, 10, and 11 (BA103) is shown in (Figure 2). The 1,2,4 triazole ring of **4** is involved in hydrogen bond interactions with Thr323, while the urea NH groups bind the backbone of Pro274. A cation–π interaction involves the triazole ring and Arg276. The triazole ring of 10 forms a hydrogen bond with the side chain of Arg272, while the pyridine is involved in two hydrogen bond interactions with the same amino acidic residue. The urea NH groups form two hydrogen bonds with the backbone of Pro274. The triazole moiety of BA103 establishes hydrogen bonds with Arg276, Arg326, and Thr323, while the two urea NH groups bind the carbonyl group of the backbone of Pro274.

**Figure 3:**
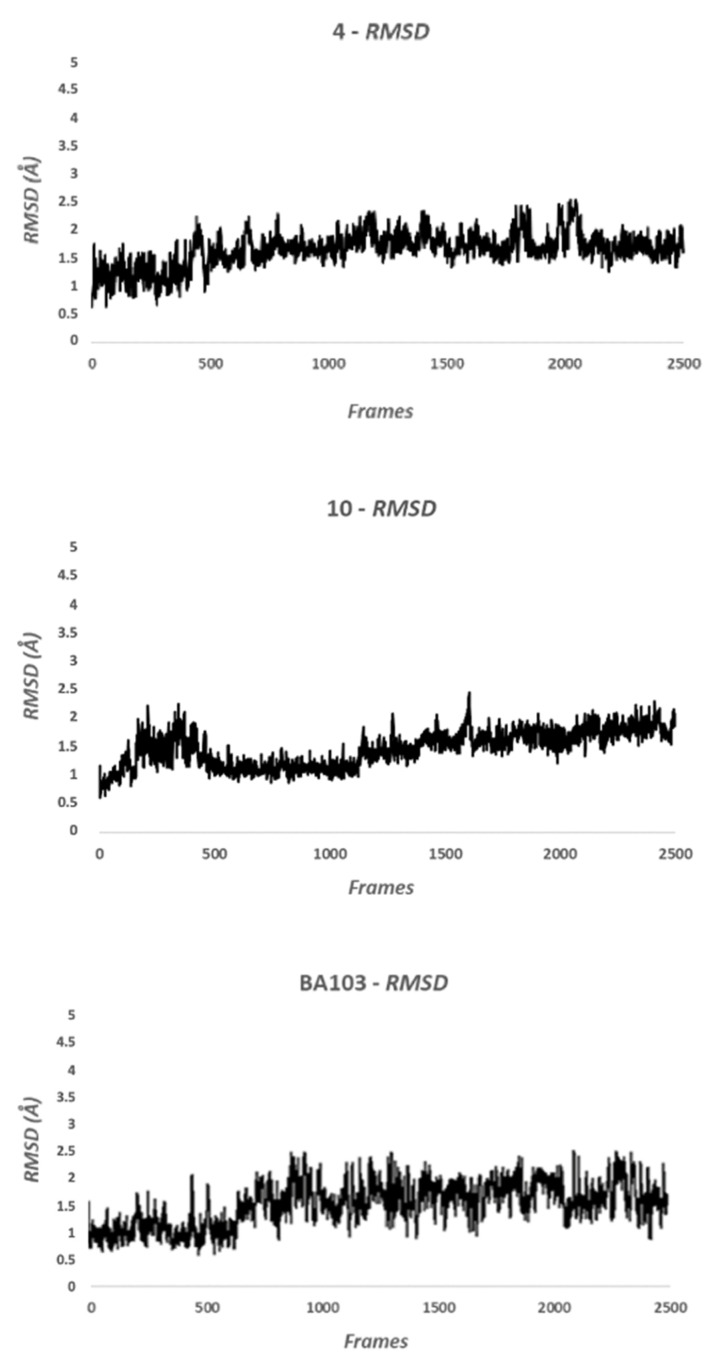
Root Mean Square Deviation of 4, 10, and BA103 during the Molecular Dynamic simulations. Values below to 3Å indicating that compounds stably interact with residues that constitute the binding site for the whole simulation.

After docking calculation, 100 ns of molecular dynamic (MD) simulation was performed using Amber 16 [31]. The Root Mean Square Deviation (RMSD) analysis showed that the compounds **4**, **10**, and **BA103** did not modify their position on the binding site, highlighting the stability of the complex. The RMSD value was less than 2Å, confirming the visual analysis hypothesis (Figure 3). BA103 maintained essential contacts with the protein for the whole simulation. The most important interactions were three hydrogen bonds with the side chain of Thr323 and with the backbone of Pro274 and Arg326.

**Figure 4:**
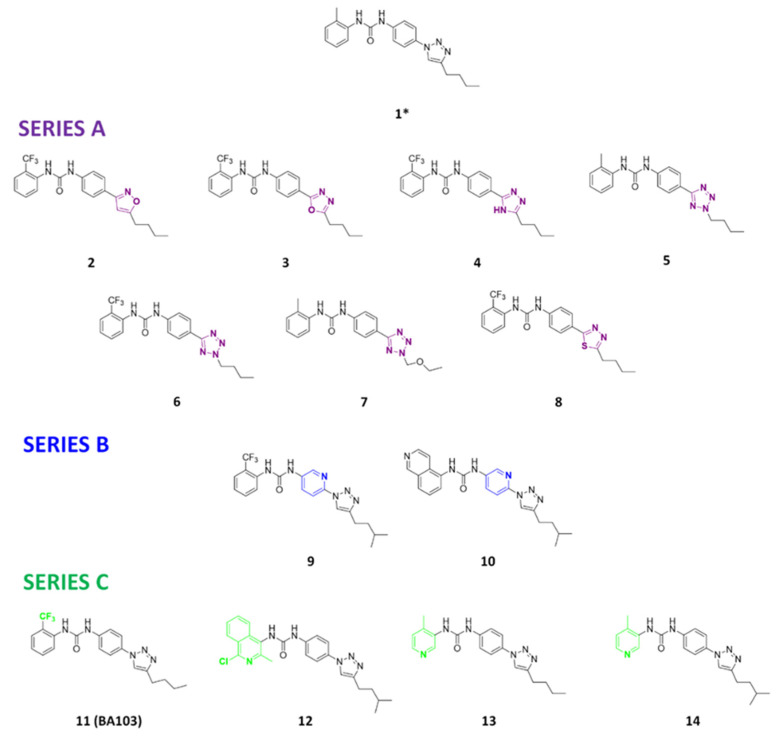
Two-dimensional (2D) structures of the hit compound already published (compound 1) and of the novel compounds selected through our computational model. * Hit compound previously published.

Three novel series of compounds were selected to explore, in turn, the importance of triazole (series A), phenyl (series B), and tolyl (series C) moieties of the hit **1** (Figure 4). As a result, fourteen derivatives endowed with the best docking score, and predicted ADME parameters were synthesized as described below.

### 3.2. Biological Evaluation 

All the compounds were tested for their ability to inhibit the helicase activity of DDX3X using a fluorescence resonance energy transfer (FRET)-based assay, as previously described [19,32]. Values of fluorescence signal in the presence or in the absence of inhibitor molecules were analyzed by linear interpolation (for details, see Methods 2.3.2). As reported in Table 1, several derivatives showed activities in the submicromolar range, in particular compounds **4**, **6**, **10**, and **BA103** (Figure S2).

In series A, the replacement of a triazole ring with oxazole was detrimental for the solubility of compound **2**, which precipitated from medium preventing its biological evaluation. Oxadiazole (compound **3**) maintained a good activity value of 1.0 µM. Our docking analysis indeed demonstrated that **3** is able to maintain profitable interaction with all the key amino acids, including a hydrogen bond between the oxygen of the oxadiazole ring and Arg276. 1,2,4 Triazole **4** shows the best inhibitory activity of 0.07 µM. Docking analysis highlighted that **4** retains the interactions with the backbone of Pro274 and establishes a cation–π interaction with Arg480; in addition, the nitrogen of the triazole ring interacts with the side chain of Thr323 and forms a cation–π interaction with Arg276.

The replacement of triazole with tetrazole is typically used to increase the permeability [33] and consequently the biodistribution of compounds. In our case, the binding poses of the tetrazole compounds revealed an additional hydrogen bond between the nitrogen of the tetrazole ring and Arg276; however, only the central phenyl ring of **6** establishes a cation–π interaction with Arg480. Accordingly, this modification affects the inhibitory activity of **5,** which is 16 times less active than the hit compound **1**; in contrast, **6** maintains a good activity of 0.8 µM, which is probably due to the presence of the trifluoromethyl group, which already increased the potency of DDX3X inhibitors characterized by sulfonamide scaffold [26]. According to our previous findings [26], we replaced the butyl chain of **6** with the ethoxymethyl side chain, but the activity decreased to 14 µM (compound **7**). Finally, the replacement with 1,3,4-thiadiazole was detrimental for the activity of **8**, which shows an IC_50_ value of 50 µM.

In series B, a pyridine nucleus was introduced in the central ring to increase the solubility of our derivatives; according to in silico study, the butyl was replaced with an isopropyl side chain. This modification slightly decreased the enzymatic activity of **9**. In contrast, the corresponding isoquinoline derivative **10** showed a good activity, confirming the importance of the isoquinoline portion that we already observed for other DDX3X inhibitors [26]. Our in silico evaluation highlighted that the pyridine nitrogen of **9** interacts with Arg276; while derivative **10** establishes important interactions within the pocket such as two hydrogen bonds between the pyridine nitrogen and Arg276, one hydrogen bond between Arg276 and the triazole ring, and two hydrogen bonds between the urea and the Pro274 backbone. In order to check the importance of trifluoromethyl and the isoquinolinyl portion in the triazole series (series C), we synthesized compounds **11** (**BA103**) and **12**, respectively. The trifluoromethyl group maintains the inhibitory activity of **BA103** comparable with the hit **1** (0.3 µM). The replacement of tolyl terminus with 1-chloro-3-methylisoquinolin-4-yl or 4-methylpyridine-3-yl rings, instead, resulted in the loss of activity from five to 33 times (compounds **12**, **13**, **14**).

We previously demonstrated that compound **1** specifically inhibits the helicase activity of DDX3X by competing with RNA substrate, excluding a direct inhibition of its ATPase activity [22,24]. To confirm the proposed mechanism of action and the selectivity of our compounds for the helicase binding site, we tested the capability of **BA103** to inhibit the ATPase activity of DDX3X and the helicase activity of DDX1, which is another human member of the DEAD-box family. As shown in Table 2, no inhibition of the ATPase activity of DDX3X was found with any compound concentration, confirming the selectivity of our compounds for the RNA pocket. Analogously, compounds **1**, **4**, and **10** were completely unable to inhibit the helicase activity of DDX1.

**Figure 5:**
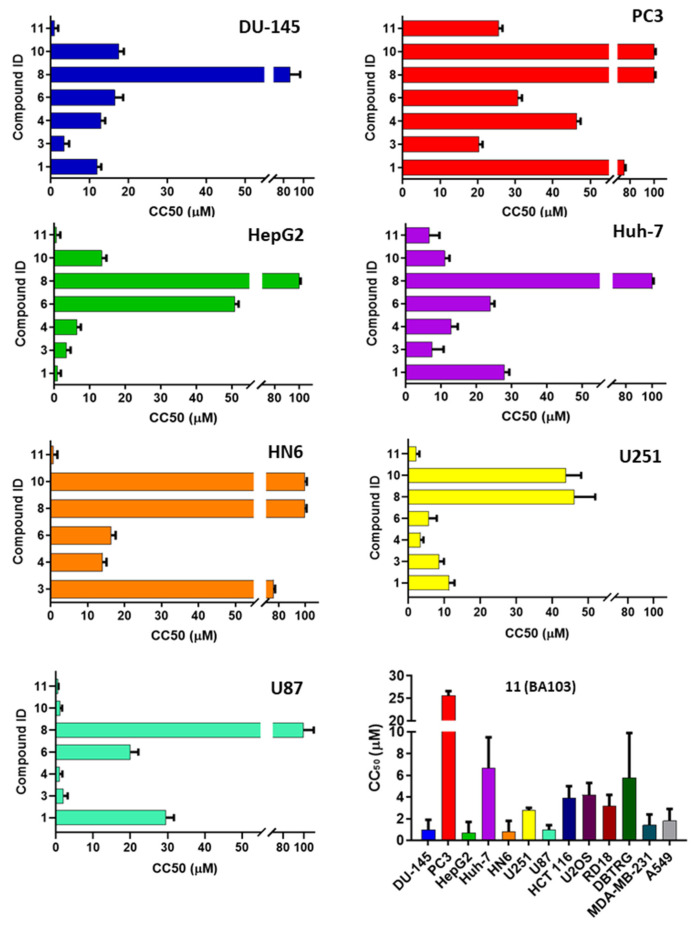
Cytotoxicity of selected compounds. Compounds were assayed on DU-145, PC3, HepG2, Huh-7, HN6, U251, and U87 cell lines. The concentration of the compound, which reduced the number of viable cells by 50% (CC_50_), was determined by dose–response curve, and the mean CC_50_ value for each cell line was calculated by triplicate assays (raw data from U87 and U251 cells with dose–response curves used for the calculation of CC_50_ in Table S1 and Figure S2). The most active compound, **BA103**, was assayed on a panel of seven additional cancer cell lines.

Then, all compounds with an IC_50s_ lower than 1 μM were screened against a panel of cancer cell lines, including prostate (Du-145, PC3), hepatic (HepG2, Huh-7), head and neck (HN6) cancer cells, and GBM cells (U87 and U231). Cells were incubated with a scalar concentration of DDX3X inhibitors, and after 72 h of treatment, cell viability was assessed by cytotoxicity assay. As reported in Figure 5, compound **BA103** showed the best anticancer profile, with CC_50s_ in the low micromolar range against DU-145, HepG2, HN6, and U87. The most potent derivative against the DDX3X enzyme, compound **4**, showed inhibition growth capability in all the cell lines tested, but unfortunately, it was lower than expected from its CC_50_ value (Table 1), which was probably due to poor cellular permeability. These results suggest a cell type-dependent activity of the compounds, as clearly demonstrated by a comparison of cells also from the same histology origin (PC3 vs. DU-145, HepG2 vs. Huh-7, U87 vs. U231). These data are in agreement with previously published studies by Raman et al. [28]. As expected, compound **8** endowed with the lowest DDX3X inhibitory effect was poorly effective against all the cell lines tested. Compounds **4**, **6**, and **BA103** were also evaluated against BJ primary fibroblasts; compound **4** has a cytotoxicity value of 84.7 μM; on the contrary, compounds **6** and **BA103** were not toxic up to 200 μM concentration. This further excluded a possible nonspecific cytotoxic effect. In this respect, compound **BA103** showed inferior toxicity against BJ cells than as previously reported for RK-33 [15]. Due to its interesting profile, compound **BA103** was further tested against additional pulmonary (A549), breast (MDA-MB-231), colorectal (HCT-116), osteosarcoma (U2OS), rhabdomyosarcoma (RD18), GBM (DBTRG, U87, and U251) and medulloblastoma (DAOY) cancer cell lines. As reported in Figure 5, **BA103** confirmed its broad-spectrum inhibition growth, showing micromolar CC_50s_ against the nine additional cell lines tested.

It has been proposed that the anticancer effect of compound RK-33 is correlated with the DDX3X expression levels [9]; thus, we aimed to verify this aspect for our compounds also, and we quantified the DDX3X protein concentrations in selected cell lines. Data were collected in Table 3. Compound **BA103** was more effective in cell lines with higher DDX3X levels (DU-145 and HepG2). However, this trend was not confirmed in all the cell lines. Thus, it is likely that sensitivity to DDX3X inhibition does not only depend on its expression levels but also on the genetic background associated with a specific cancer phenotype, which is in agreement with the multifaceted roles of DDX3X in tumor transformation [29]. Nevertheless, further studies should be performed to exclude possible interactions with other cellular targets.

**Figure 6:**
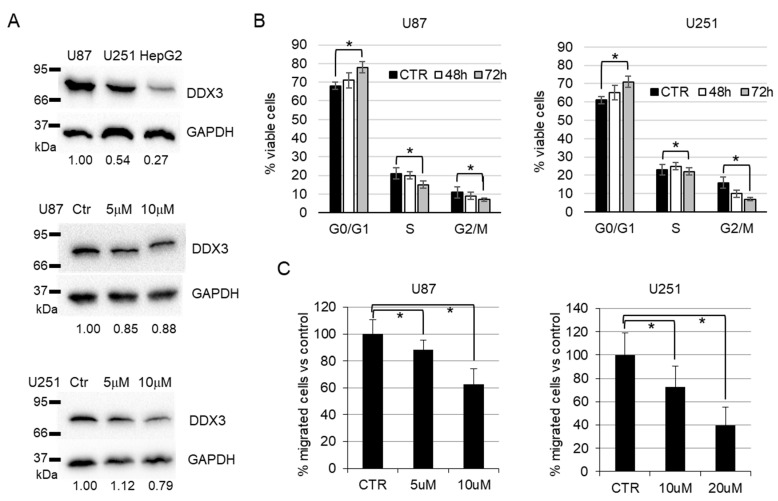
In vitro biological activity of **BA103** in GBM U87 and U251 cells. (**A**) DDX3X protein expression in whole-cell lysate of U87 and U251 cells: DDX3X expression was evaluated in comparison with cell lysate from HepG2 (upper blots) and after 24 h of treatment with BA103. Expression of GAPDH was evaluated as the loading control, and relative densitometry for DDX3X band expression is indicated at the bottom of the blots. Detailed information about Western blot can be found in the Supplementary Materials (Figure S3). (**B**) Cell cycle analysis of U87 and U251 cells treated for 48 h and 72 h with 10 µM **BA103**. Result is expressed as the mean percentage of cells in each cell cycle phase with respect to the total number of analyzed cells. Three different experiments were considered. (**C**) The migration ability of U87 and U251 cells was evaluated after 48 h of incubation with **BA103** at the indicated concentrations, and results were expressed as the percentage with respect to vehicle-treated control cells. The number of migrated cells was the mean value of three different experiments. * *p* < 0.01 according to Student’s t-test with respect to the control value, as indicated by the bracket.

**Figure 7:**
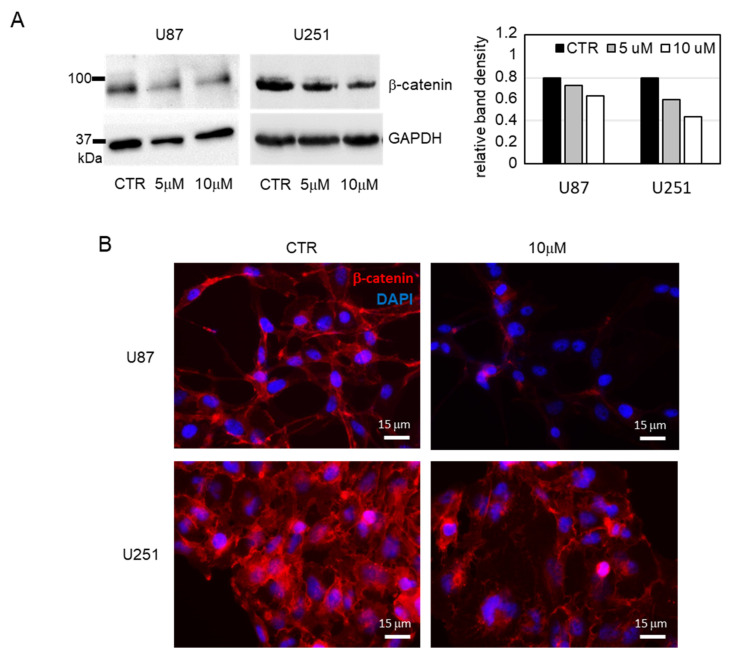
Expression of β-catenin protein in GBM U87 and U251 cells treated with **BA103**. (**A**) β-catenin expression was analyzed by Western blot in the cell lysate of GBM cells treated for 48 h with 1 and 10 μM **BA103**. The expression level of GAPDH is shown as the loading control, and the analysis of relative band density is reported in the histogram on the right. Detailed information about Western blot can be found in the Supplementary Materials (Figure S3). (**B**) β-catenin expression was analyzed by immunofluorescence in GBM U87 and U251 treated with 10 μM **BA103** for 48 h. Nuclei were stained with DAPI. Images were acquired by fluorescence microscope at 400× magnification.

To better characterize the antiproliferative effect of **BA103**, we performed further biological evaluation using GBM cell lines. The expression of DDX3X was evaluated in U87 and U251 cell lines by Western blot analysis in comparison with HepG2 cells, resulting in basally detectable mainly in U87 cells (Figure 6A). The treatment of GBM cells with **BA103** determined a reduction in the expression of DDX3X (Figure 6A) and significant accumulation of cells in the G0/G1 phase of the cell cycle after 72 h (Figure 6B). Cell cycle experiments were performed in normal conditions (CTR) and in the presence of **BA103** at 1 µM and 10 µM concentrations for 48 h and 72 h. These results are in agreement with silencing experiments reported by Lai et al. [30], and they are consistent with the cell cycle block previously reported for RK-33 treatment [28]. In addition, after 48 h of incubation with **BA103**, GBM cells demonstrated a reduced ability to migrate through collagen-coated Transwell filters with respect to untreated cells (Figure 6C). Previous studies have demonstrated that DDX3X regulated β-catenin protein expression through the stabilization of Rac1 mRNA translation in a helicase-dependent manner [34]. Thus, also according to the importance of β-catenin signaling in GBM, we analyzed whether the expression of β-catenin in U87 and U251 cells could be associated with DDX3X activity. GBM cells were treated with **BA103** for 48 h, and then, β-catenin expression was evaluated by Western blot analysis and immunofluorescence analysis (Figure 7A,B, respectively). In both cell lines, we observed a reduction in the expression of β-catenin after **BA103** treatment, which was more evident in U87 cells.

### 3.3. In Vitro ADME 

**Figure 8:**
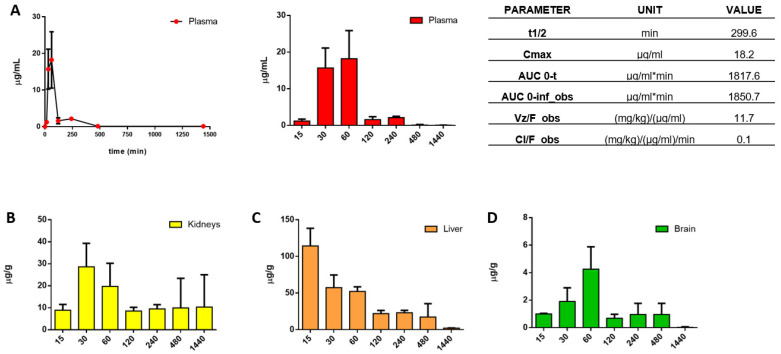
PK and biodistribution of **BA103** in BALB/c mice at the single dose of 50 mg/kg i.p. (**A**) Plasmatic distribution and PK parameters. Concentration levels ± SD of **BA103** in (**B**) kidneys, (**C**) liver, and (**D**) brain.

Preliminary in vitro ADME analysis was performed to early evaluate the pharmacokinetic (PK) profile of our compounds (Figure 8). Aqueous solubility was calculated by LC-MS-MS analysis. An aliquot of 1 mg of compound was stirred for 24 h at 27 °C in 1 mL of water. The mixture was filtered, and the quantity of solubilized compound was determined. Passive permeability was determined using parallel artificial membrane permeability assay (PAMPA). Microsomal metabolic stability was determined by LC-UV-MS analysis by incubating compounds with human liver microsomes at 37 °C for 60 min (for details, see the Supporting Information).

As shown in Table 4, all selected derivatives possessed optimal metabolic stability values ranging from 89 to 97%. Derivative **4** showed a little improvement of its aqueous solubility with respect to compound **1** (logS = −6.56 vs. −7.05), but it possessed very low passive permeability, which can be the cause of its modest anticancer activity. On the other hand, oxazole **3**, tetrazole **6**, and **BA103** showed good values of passive permeability, which make them suitable candidates also for oral administration.

### 3.4. In Vivo Experiments 

Thus, preliminary PK and biodistribution studies were performed on two groups of BALB/c mice (CTRL and **BA103**-treated) at the single intraperitoneal (i.p.) dose of 50 mg/kg. **BA103** was slowly eliminated from plasma, with a half time (t1/2) of about 5 h. A higher concentration of compound **BA103** was found in the liver followed by kidney, plasma, and brain (Figure 8), confirming its ability to cross the blood–brain barrier (BBB). Even if the PK analysis was conducted in healthy mice, with an intact BBB, the data are of great importance, since the majority of drugs are unable to reach the tumor environment due to their low permeability.

**Figure 9:**
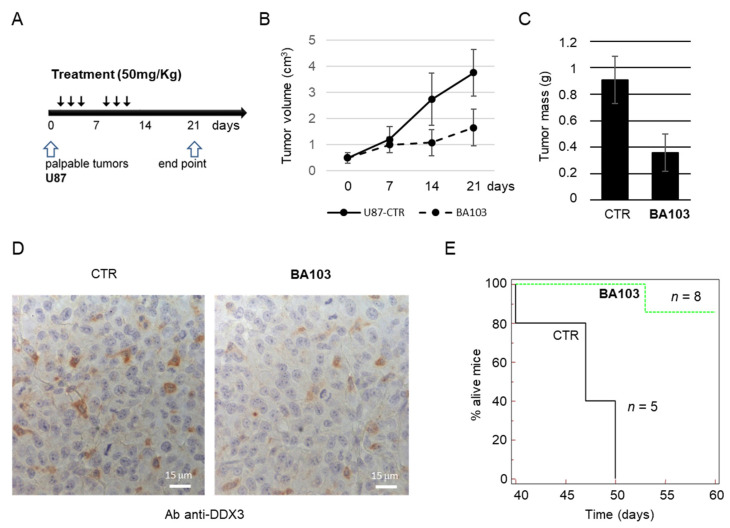
Biological activity of **BA103** in xenograft models of GBM. (**A–D**) Mice were inoculated s.c. with U87 cells, randomly distributed in two groups (CTR and **BA103**), and treated according to the protocol shown in panel (**A**). Tumor growth was monitored by measuring tumor volume (**B**), and at the end point, tumors were weighed, and the resulting mean values for each group are reported in the histogram (**C**). Recovered tumor masses were processed for analysis of DDX3X expression by immunohistochemistry, and representative images acquired at 400× magnification are shown in panel (**D**). (**E**) Survival curve from in vivo orthotopic model of GBM. Mice were divided in two groups, control (CTR, *n* = 5) receiving vehicle, and inhibitor-treated (*n* = 8) receiving an i.p. injection of 50 mg/kg **BA103**. Mice were monitored for two months after U87 cells inoculation.

Next, we tested our compound on U87 cells in a xenograft model of GBM. First, U87 cells were inoculated subcutaneously into 16 male immunodeficient mice, and tumors were allowed to grow until they reached a palpable size. Then, mice were randomly assigned to one of the two experimental groups: vehicle-treated group (control) or drug-treated group (receiving 50 mg/kg **BA103**). The treatment protocol is depicted in Figure 9A and included three i.p. administrations per week for two consecutive weeks. During the experiment, tumor growth was monitored measuring tumor volume, and at the end point, 21 days after the start point, mice were euthanized, and tumors were recovered and measured. A reduction in tumor growth was evident in the treated group with respect to the control group as early as from the second week (Figure 9B). At the end point, mice treated with DDX3X inhibitor showed a significant reduction of mean tumor mass, with about a 60% reduction with respect to the mean tumor mass of the control group (Figure 9C). Tumor masses were also analyzed for the expression of DDX3X, and all xenografts resulted positive for DDX3X expression, from both control and treated groups, with a spotted staining pattern (Figure 9D).

Given the good biodistribution of our compound into the brain, we also performed an orthotopic xenograft experiment inoculating intracerebrally U87 cells in immunodeficient mice, and randomizing mice 1 week after inoculation in the control group (five mice, vehicle) and treated group (eight mice, 50 mg/kg **BA103**). Mice received three i.p. administrations per week, for three consecutive weeks, and then, they were observed for two months. As shown by the survival curve in Figure 9E, at the endpoint, seven out of eight mice in the **BA103**-treated group were alive, whereas all mice in the control group died within the 50th day after the start of the surveillance.

## 4. Discussion

Cancer remains one of the main causes of mortality and morbidity worldwide, making the development of novel molecular targeted anticancer drugs an urgent need, yet it remains a relevant challenge for the scientific community. Indeed, effective drugs are lacking for many malignancies, such as GBM, the most aggressive brain cancer that, from a pharmaceutical point of view, can still be considered an orphan disease.

DEAD-box proteins represent interesting targets for the development of antiviral and anticancer compounds. In particular, in the last few years, a growing number of studies demonstrated a role of the DEAD-box RNA helicase DDX3X in different mechanisms related to tumor proliferation, including cell cycle progression, apoptosis, hypoxia response, and migration, further corroborating its suitability as an anticancer drug target [9,10,11]. Starting from our long-standing expertise in developing small-molecule DDX3X inhibitors, herein, we disclose the synthesis of new DDX3X helicase inhibitors characterized by different heterocyclic moieties. We discovered novel more potent derivatives and new important chemical features that could be modified to further improve both the enzymatic and cellular activities of our inhibitors. DDX3X acts as an oncogene through the deregulation of the Wnt/β-catenin pathway or increasing Snail expression [12]. The correlation between DDX3X and Snail expression has been reported in different cancer types, and it is correlated with a poor prognosis [13]. In tumors with non-functional p53, DDX3 inhibits DNA damage-induced caspase activation, promoting tumor growth [14].

Based on these findings, several small molecule inhibitors of DDX3X have been developed as anticancer agents even if designed to target the ATPase activity of the enzyme. Among them, RK-33, NZ51, and Ketorolac were able to reduce tumor volume in xenograft Ewing Sarcoma [15], in preclinical lung and prostate cancer models in association with radiotherapy, and in a tongue tumor mouse model [16], furnishing a proof of concept in vivo. Starting from literature studies, we screened the most interesting derivatives in a panel of cancer cell lines, and we identified compound **BA103** endowed with low micromolar CC_50s_ against fourteen cancer cell lines that includes prostate, hepatic, head and neck, and GBM (DU-145, HepG2, HN6, U87, DBTRG, and U251). Even if data were of importance against multiple cancer subtypes, in particular hepatocarcinoma, we focused our attention on GBM, which is an orphan disease characterized by poor prognosis. To date, the life expectancy span is 1 to 2 years, and the treatment is represented by radio/chemotherapy followed by the surgical removal of tumor mass [23]. Even if the role of DDX3X in human gliomas is not completely understood, its expression is significantly higher in glioma cells compared to normal brain tissue [35]. In addition, as highlighted by Sun et al. [13], DDX3X regulates Snail expression in several cancer types, including GBM, increasing cell migration and promoting GBM progression.

Compound **BA103** blocked U87 and U251 cells in the G1 phase, which is similar to previously published results for ATPase DDX3X inhibitors on different tumor cell lines.

In vitro ADME analysis of the most active compounds showed values of aqueous solubility outside the recommended range; nevertheless, several derivatives possess good metabolic stabilities and high values of passive permeability, making them suitable candidates for oral administration in further in vivo experiments. Preliminary biodistribution studies confirmed that compound BA103 reached all organs and, most importantly for the purpose of this study, the brain.

When evaluated in a murine xenograft GBM model, compound BA103 administered for two consecutive weeks was able to significantly block tumor growth in the absence of any visible sign of distress in the receiving animal. An effective inhibitory effect was achieved for both subcutaneous large tumors and early brain tumors, determining a significant reduction in tumor mass and a marked increase in survival, respectively. In addition, the capacity to inhibit tumor growth in the intracranial model indirectly confirmed the ability of BA103 to pass the altered BBB typical of GBM. In vitro data suggest that these effects were determined not only by a block in cell cycle progression but also by the inhibition of other tumor-promoting functions, including migration ability. This hypothesis is corroborated by the observed reduction of β-catenin protein levels in GBM cell lines treated with **BA103**. That β-catenin stability is associated with DDX3X activity has been already demonstrated by other authors in different experimental models [34,36]. These results provide the first evidence that BA103 is effective in blocking tumor progression in a monotherapy protocol and not only as adjuvant treatment, as previously suggested for a different DDX3X inhibitor used in combination with radiotherapy [31]. Further studies are ongoing to confirm the in vivo antitumoral potency of our compound also against other cancer subtypes such as hepatocarcinoma.

## 5. Conclusions

In conclusion, our study demonstrates for the first time the feasibility of using inhibitors of the helicase-binding site of DDX3X not only as broad-spectrum antivirals but also as new potent broad-spectrum targeted anticancer compounds that are active against difficult to treat tumors such as GBM.

## Figures and Tables

**Table 1:** DDX3X anti-enzymatic activity.

Cpd. ID	IC_50_ ± SD(μM) ^[a,b]^
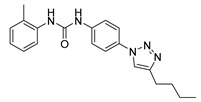 **1** ^[c]^	0.30 ± 0.07
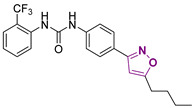 **2**	nd ^[d]^
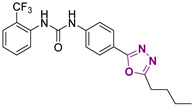 **3**	1.0 ± 0.2
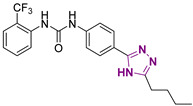 **4**	0.07 ± 0.02
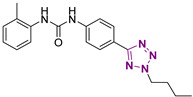 **5**	4.9 ± 0.5
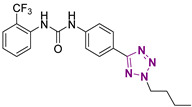 **6**	0.8 ± 0.2
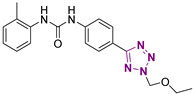 **7**	14 ± 2.0
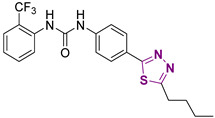 **8**	50 ± 7
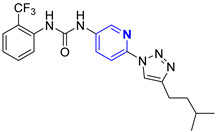 **9**	1.1 ± 0.2
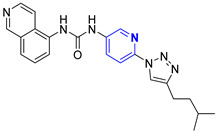 **10**	0.10 ± 0.03
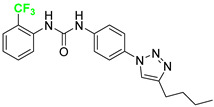 **11/BA 103**	0.40 ± 0.05
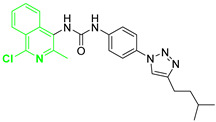 **12**	10 ± 1.0
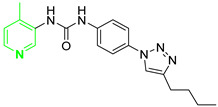 **13**	1.5 ± 0.3
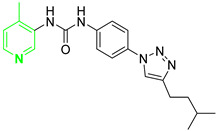 **14**	2.0 ± 0.3

^[a]^ Data represent the mean of at least two independent experiments. ^[b]^ IC_50_: inhibiting concentration 50 or needed concentration to inhibit 50% of the enzyme. ^[c]^ previously published compound ^[d]^ nd, not determined, compound precipitated during the assay.

**Table 2:** Selectivity data on selected compounds.

Cpd ID	ATPase DDX3X IC_50_ ± SD, µM	DDX1 IC_50_ ± SD, µM	SI
**1** *	>200 ^[a]^	>200 ^[a]^	>666
**4**	NT	29.29 ± 16.75	418
**10**	NT	>100	>1000
**BA103**	>200 ^[a]^	>100	>250

^[a]^ The value >200 indicates that less than 20% of inhibition was observed at 200 µM, the highest concentration tested. * 1 was used as a reference compound. NT: not tested. SI Selectivity Index, calculated as the ratio between the helicase activity (IC_50_) against DDX3X and DDX1. Data represent the mean of two values of the least three experiments.

**Table 3:** Concentration of DDX3X per cell ^[a]^.

Cell Line	U2OS	A549	Huh-7	DU-145	HepG2
DDX3X ± SD (nM)	174 ± 20	103 ± 10	755 ± 75	372 ± 20	538 ± 33

^[a]^ Values represent mean ± S.D. of three independent experiments. Data represent the mean value of the least three independent experiments. For details, see Methods.

**Table 4:** In vitro ADME evaluation of selected compounds ^[a]^.

Cpd. ID	AppP ^[b]^	Memb. ^[c]^ Ret. %	LogS ^[d]^	HLM Stability ± SD ^[e]^
**1**	2.86 × 10^−6^	19.1	−7.05	99.0 ± 0.6
**3**	9.75 × 10^−6^	10.3	<−7.60	88.9 ± 0.9
**4**	<0.1 × 10^−6^	2.3	−6.56	97.9 ± 0.3
**6**	10.23 × 10^−6^	21.6	<−7.60	90.32 ± 0.7
**10**	1.21 × 10^−6^	11.3	<−7.44	91.31 ± 0.1
**11/BA103**	7.47 × 10^−6^	23.9	−7.43	97.0 ± 0.5

^[a]^ Values represent the mean values of three independent experiments. [^b^] Apparent permeability reported in cm·s^−1^. ^[c]^ Membrane retention %. ^[d]^ Aqueous solubility. ^[e]^ Human Liver Microsomal Metabolic Stability.

## Data Availability

The data presented in this study are available in Supplementary Information.

## Supplementary Materials

The following are available online at https://www.mdpi.com/article/10.3390/cancers13215569/s1, Figure S1: Binding mode of selected compounds; Table S1: Raw data of optical absorbance at 490 nm from MTS assay in U87 and U251 cells; Figure S2: CC_50_ curves for U87 and U251 cell lines; Figure S3: Uncropped blots; Scheme S1. Synthesis of oxazole 2; Scheme S2. Synthesis of oxadiazole 3; Scheme S3. Synthesis of triazole 4; Scheme S4. Synthesis of tetrazoles 5-7; Scheme S5. Synthesis of thiazole 8; Scheme S6. Synthesis of triazoles 9 and 10; Scheme S7. Synthesis of triazoles 11-14; Full synthetic Procedures; In vitro ADME assays.
