# Supplementary material for: Targeting DDX3X Helicase Activity with BA103 Shows Promising Therapeutic Effects in Preclinical Glioblastoma Models
Source: Cancers (Basel). 2021 Nov 7;13(21):5569. doi: 10.3390/cancers13215569 (PMC8582824; doi:10.3390/cancers13215569)
Supplement: Supplementary file 1 [file cancers-13-05569-s001.zip › cancers-1417654-supplementary.pdf]

Supporting Information

Table of contents

Figure S1. Binding mode of selected compounds 3

Table S1: Raw data of optical absorbance at 490 nm from MTS assay in U87 and U251 cells........ 4

Figure S2. CC_{50} curves for U87 and U251 cell lines. 5

Figure S3 Uncropped blots 6

Synthetic procedures 7

In vitro ADME assays 19

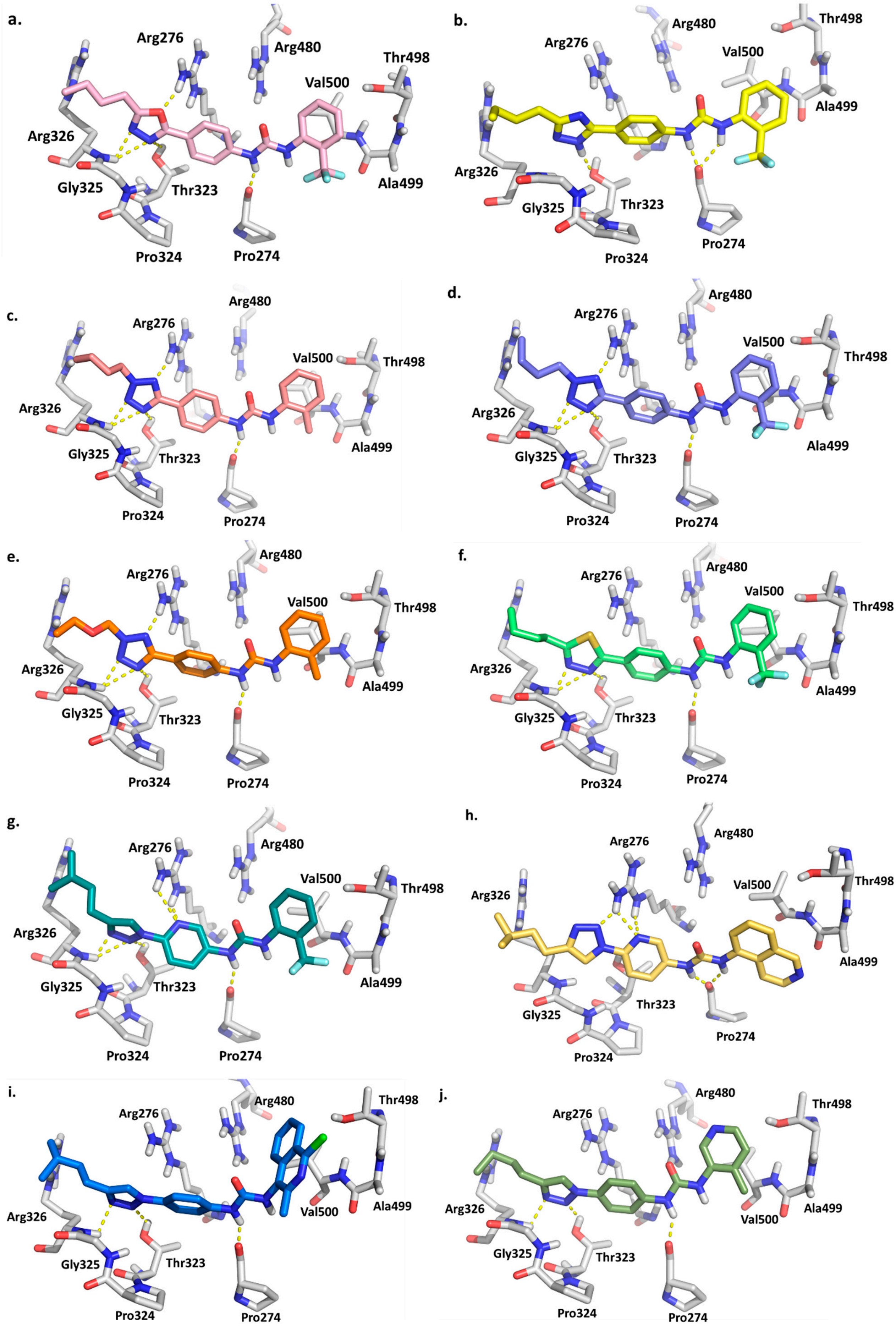
a.
Arg276
Arg480
Thr498
Val500
Arg326
Gly325
Thr323
Ala499
Pro324
Pro274
b.
Arg480
Thr498
Arg276
Val500
Ala499
Arg326
Gly325
Thr323
Pro274
Pro324
c.
Arg480
Arg276
Val500
Thr498
Arg326
Ala499
Gly325
Thr323
Pro324
Pro274
d.
Arg480
Arg276
Val500
Thr498
Arg326
Ala499
Gly325
Thr323
Pro324
Pro274
e.
Arg480
Arg276
Val500
Thr498
Arg326
Ala499
Gly325
Thr323
Pro324
Pro274
f.
Arg480
Arg276
Val500
Thr498
Arg326
Ala499
Gly325
Thr323
Pro324
Pro274
g.
Arg480
Arg276
Val500
Thr498
Arg326
Ala499
Gly325
Thr323
Pro324
Pro274
h.
Arg480
Arg276
Arg326
Val500
Thr498
Ala499
Thr323
Gly325
Pro274
Pro324
i.
Arg480
Thr498
Arg276
Arg326
Val500
Ala499
Gly325
Thr323
Pro324
Pro274
j.
Arg480
Thr498
Arg276
Arg326
Ala499
Val500
Gly325
Thr323
Pro324
Pro274

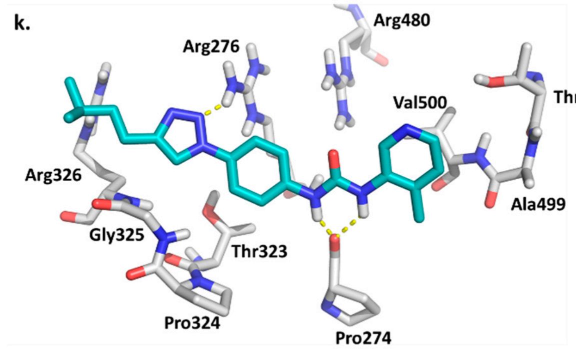

Figure S1. Binding mode of selected compounds.

a. Binding mode of **3**. The oxygen atom of the oxadiazole ring interacts with Arg276, while the two nitrogen atoms are involved in hydrogen bond interactions with Gly325 and with the side chain of Thr323. Pro274 binds the urea NH-groups. **b.** Binding mode of **4**. The 1,2,4 triazole ring of 4 is involved in hydrogen bond interactions with Thr323 while the urea NH groups bind the backbone of Pro274. A cation-π interaction involves the triazole ring and Arg276. **c. d. e.** Binding mode of **5, 6** and **7**. The tetrazole rings of **5, 6** and **7** establish hydrogen bond interactions with the side chains of Arg276 and Thr323 and with Gly325. The urea moiety binds the backbone of Pro274. A cation-π interaction involves the central aromatic ring of **6** and **7** and Arg276. **f.** Binding mode of **8.** The 1,3,4-thiadiazole ring establishes hydrogen bond interactions with Gly325 and with the side chain of Thr323, while the urea is involved in hydrogen bond interactions with Pro274. A cation-π interaction involves the central phenyl ring and Arg276. **g.** Binding mode of **9**. The triazole ring interacts with Gly325 and with the side chain of Thr323. The pyridine ring forms two hydrogen bonds with Arg276, while the urea moiety interacts with the backbone of Pro274. **h.** Binding mode of **10**. The triazole ring of **10** forms a hydrogen bond with the side chain of Arg272, while the pyridine is involved in two hydrogen bond interactions with the same amino acidic residue. The urea NH-groups form two hydrogen bonds with the backbone of Pro274. **i.** Binding mode of **12**. The triazole ring of **12** interacts with Gly325 and with the side chain of Thr323. The urea moiety interacts with the backbone of Pro274. A cation-π interaction is formed between the triazole ring and Arg276. **j.** Binding mode of **13**. The triazole ring of compound **13** establishes hydrogen bond interactions with Gly325 and Thr323, while the urea group is involved in interactions with Pro274. A cation-π interaction is formed between the triazole ring and Arg276. **k**. Binding mode of **14**. The triazole ring of 14 binds the side chain of Arg276. The urea NH-groups form two hydrogen bond interactions with the backbone of Pro274.

Table S1: Raw data of optical absorbance at 490 nm from MTS assay in U87 and U251 cells. Tables contain means with SD of at least 5 different values that were used for preparation of figure 5.

U87 (mean absorbance)						
	**concentration (uM)**					
**compound**	**0**	**0,01**	**0,1**	**1**	**10**	**100**
**1**	2,035	2,027	2,018	2,000	1,584	0,465
**3**	2,026	1,350	1,140	1,178	1,065	0,568
**4**	2,010	1,906	1,293	0,941	0,784	0,162
**6**	1,943	1,944	1,964	2,007	1,387	0,540
**8**	2,456	2,267	1,994	1,965	1,945	1,054
**10**	2,141	1,863	1,379	1,165	0,858	0,467
**11**	2,024	1,598	1,132	0,959	0,804	0,566
U87 (SD absorbance)						
	**concentration (uM)**					
**compound**	**0**	**0,01**	**0,1**	**1**	**10**	**100**
**1**	0,015	0,018	0,015	0,125	0,125	0,083
**3**	0,065	0,119	0,172	0,124	0,263	0,040
**4**	0,130	0,066	0,536	0,026	0,057	0,006
**6**	0,259	0,049	0,150	0,188	0,035	0,225
**8**	0,167	0,077	0,067	0,168	0,052	0,078
**10**	0,164	0,057	0,059	0,119	0,050	0,061
**11**	0,217	0,158	0,025	0,024	0,041	0,041

U251 (mean absorbance)						
	**concentration (uM)**					
**compound**	**0**	**0,01**	**0,1**	**1**	**10**	**100**
**1**	2,019	2,020	1,998	1,459	1,107	0,474
**3**	2,015	1,298	1,025	1,098	0,593	0,217
**4**	2,441	1,499	1,513	1,627	1,482	0,502
**6**	2,308	1,431	1,763	2,172	1,348	0,361
**8**	2,294	2,363	2,282	2,177	1,806	0,786
**10**	2,181	1,904	2,003	1,581	1,667	0,809
**11**	2,327	2,166	2,070	1,364	0,842	0,604
U251 (SD absorbance)						
	**concentration (uM)**					
**compound**	**0**	**0,01**	**0,1**	**1**	**10**	**100**
**1**	0,059	0,012	0,004	0,115	0,103	0,180
**3**	0,051	0,204	0,738	0,109	0,068	0,114
**4**	0,219	0,581	0,262	0,189	0,588	0,216
**6**	0,054	0,410	0,500	0,784	0,312	0,114
**8**	0,083	1,189	0,958	0,604	0,504	0,474
**10**	0,213	0,174	0,155	0,158	0,683	0,113
**11**	0,336	0,132	0,053	0,013	0,123	0,058

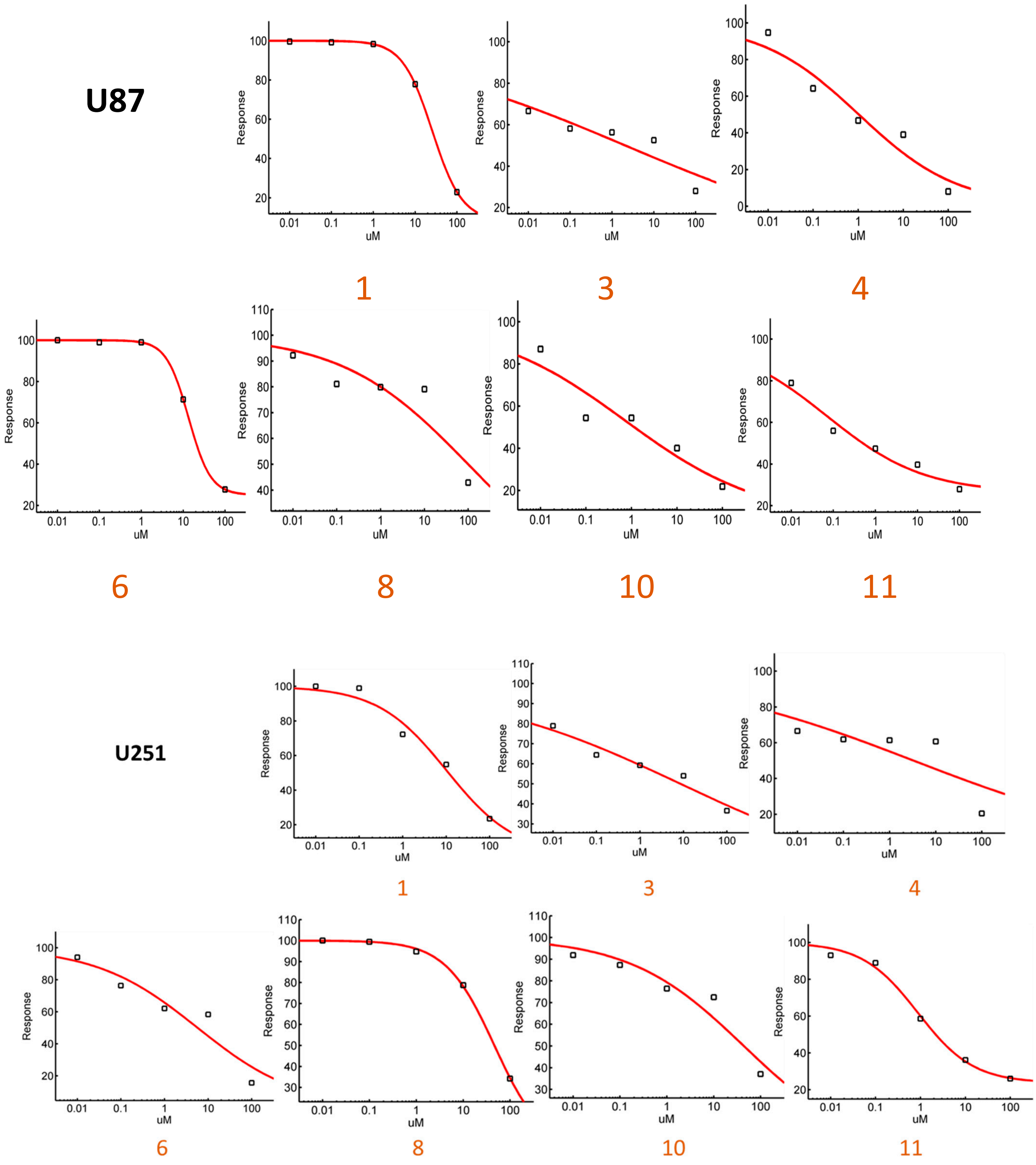

Figure S2. CC_{50} curves for U87 and U251 cell lines.
CC_{50} values were calculated with dose-response curves after plotting the absorbance data (expressed as percentage respect with control value) as a function of inhibitor concentrations and fitting the data with the software GraphPad Prism 6.0.

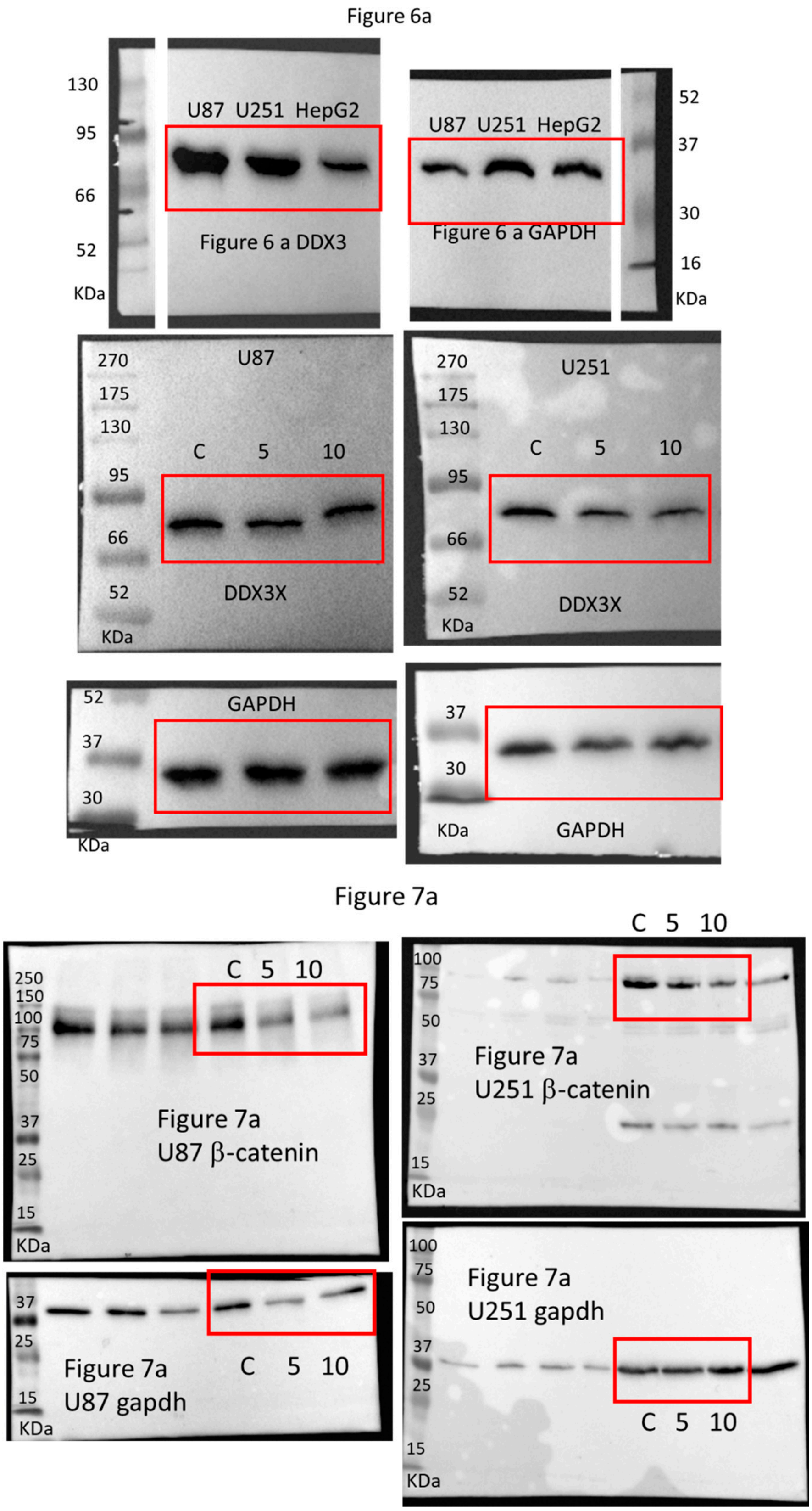

Figure S3 Uncropped blots

Digital acquisition of the whole filter of western blots used for figures 6 and 7. For each filter is included the colorimetric acquisition of the molecular weight standards

Synthetic procedures

Scheme S1. Synthesis of oxazole 2[a]

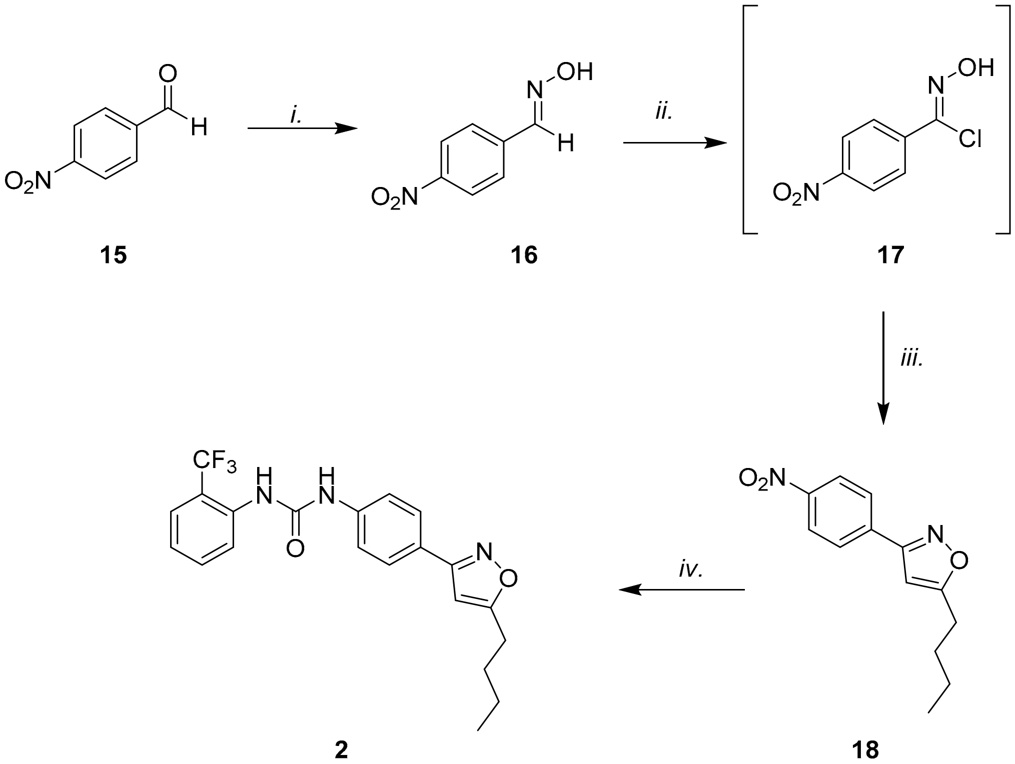

a. Reagents and conditions: *i.* $NH_2OH{\cdot}HCl$/ NaOH, EtOH/H_2O, rt., 1h (Y=99%); *ii.* N-chlorosuccinimide, hυ, DMF (dry), rt., 3h (Y=99%); *iii.* 1-hexyne, $CuSO_4{\cdot}5\,H_2O$, sodium ascorbate, H_2O *t*BuOH (1:1), $KHCO_3$, MW 80°C, 15 min. (Y=76%); *iv. a)* Zinc, DCM, CH_3COOH, rt., 7 min (Y=70%); *b)* 2-(trifluoromethyl)-phenyl-isocyanate, CH_2Cl_2 dry, rt., 12h (Y=73%).

4-Nitrobenzaldehyde oxime (16): Hydroxylamine hydrochloride (1.1 g, 15.8 mmol) was dissolved in water (10 mL) and neutralized with a 10 % NaOHaq solution. A solution of **15** (2 g, 13.2 mmol) in ethanol was added slowly to this mixture with stirring at rt for 1hr. After this time ethanol was evaporated at reduced pressure. Water was added and the rxn mixture was extracted with dichloromethane (3x40 mL). The combined organic phase was washed with brine and dried with anhydrous sodium sulfate. **16** was used for further reactions without additional purification.^{1}HNMR (400 MHz, $CDCl_3$): δ 8.25 – 8.23 (d, *J*= 7.2 Hz, 2H), 8.19 (s, 1H), 7.75 (s, 1H),7.76-7.74 (d, *J*=7.2 Hz, 2H) ppm. MS (ESI) m/z 164.9 $[M-H]^-$

N-Hydroxy-4-nitrobenzimidoyl chloride (17): 16 (100 mg, 0.60 mmol) was dissolved in dry DMF (2mL) under N_2 atmosphere. To this stirring solution, N-chlorosuccinimide was added (96.5 mg, 0.72 mmol). Initiation of the reaction was accelerated using of UV light for 20 min. After 3 hrs the mixture was poured onto crushed ice, and extracted three times with Et_2O. The organic layers were collected, dried over anhydrous Na_2SO_4 and the solvent was evaporated. **3** was used for further reactions without additional purification.

5-Butyl-3-(4-nitrophenyl)isoxazole (18): 1-hexyne (28µL, 0.25 mmol), **17** (50mg, 0.25 mmol) , $KHCO_3$ (119 mg, 1.08 mmol) were suspended in a 1:1 mixture of water and *t*-BuOH (1.5 mL each) in a 10 mL glass vial equipped with a small magnetic stirring bar. To this, was added sodium ascorbate (2 mg, 0.02 mmol) and copper(II) sulfate pentahydrate (2 mg, 0.02 mmol). The mixture was then heated for 7 min. at 80°C under microwave irradiation, using an irradiation power of 300W. After this time the solvents were partially removed, the residue was stirred with NH_4Cl ss (10mL), and NH_4OH (0.5mL) for 15 min, then extracted with EtOAc. The residue was finally purified on silica gel, to give the final product (PE/EtOAc 98:2). Yield 76%^{1}HNMR (400 MHz, $CDCl_3$): δ 8.32-8.29 (d, *J*= 8.4 Hz, 2H), 7.97-7.95 (d, *J*=8.4 Hz, 2H), 6.36 (s, 1H), 2.85-2.81 (t, *J*= 7.2 Hz, 2H), 1.77-1.70 (m, 2H), 1.46-1.41 (m, 2H), 0.98-0.94 (t, *J*= 7.2 Hz, 3H) ppm.

4-(5-Butylisoxazol-3-yl)aniline: 18 (100 mg, 0.40 mmol) was dissolved in DCM and cooled to 0°C. Zinc dust (392 mg, 6 mmol) and AcOH (366 µL) were added and the reaction mixture was stirred at rt for 30 min. After this time the mixture was filtered off on a celite pad. The pH was adjusted to 7 by addition of $NaHCO_3$ (ss), and the mixture was extracted several times. The organic layers were collected, washed with Brine and dried over anhydrous Na_2SO_4 Yield 70%. ^{1}HNMR (400 MHz, $CDCl_3$): δ 7.60-7.58 (d, *J*= 7.2 Hz, 2H), 6.72-6.70 (d, *J*= 7.2 Hz, 2H), 6.18 (s, 1H), 3.84 (bs, 2H), 2.77-2.73 (t, *J*=7.6 Hz, 3H), 1.74-1.67 (q, *J*=7.2 Hz, 2H), 1.46-1.37 (q, *J*=7.2 Hz, 2H), 0.96-0.93 (t, *J*= 7.6 Hz, 3H) ppm. MS (ESI) m/z 216.9 $[M+H]^+$

1-(4-(5-Butylisoxazol-3-yl)phenyl)-3-(2-(trifluoromethyl)phenyl)urea (2): 4-(5-butylisoxazol-3-yl)aniline (100 mg, 0.46 mmol) was added to a solution of the 1-(Trifluoromethyl)phenyl isocyanate (85 µL, 0.65 mmol) in anhydrous CH_2Cl_2 (10 mL) in one portion. The solution was stirred for 4 hours at r.t. under a nitrogen atmosphere. The solvent was removed, at reduced pressure and the residue purified by flah chromatography (PE/EtOAc 95:5) Yield 73%^{1}HNMR (400 MHz, $CDCl_3$): δ 8.00-7.98 (d, *J*=7.6 Hz, 1H), 7.71-7.69 (d, *J*=7.7 Hz, 2H), 7.58-7.48 (m, 2H*)*, 7.42-7.40 (d, *J*=7.7 Hz, 2H), 7.31-7.15 (m, 3H), 7.03 (s, 1H), 2.78-2.77 (t, *J*=6.8 Hz, 2H), 1.71-1.69 (m, 2H) , 1.44-1.40 (m, 2H), 0.96-0.93 (t, *J*=6.8 Hz, 3H) ppm ^{13}C-NMR (100 MHz, $CDCl_3$): δ 170.27, 161.47, 153.67, 138.89, 137.42, 132.41, 127.75, 127.45, 127.20, 126.67, 126.32, 123.87, 123.81, 118.79, 106.46, 29.62, 29.20, 22.18, 14.01.ppm. HRMS (ESI) m/z calcd for $C_{21}H_{20}F_3N_3O_2$ $[M - H]^-$ 402.1429, found 402.1444. HPLC Purity: 97.2%.

Scheme S2. Synthesis of oxadiazole 3[a]

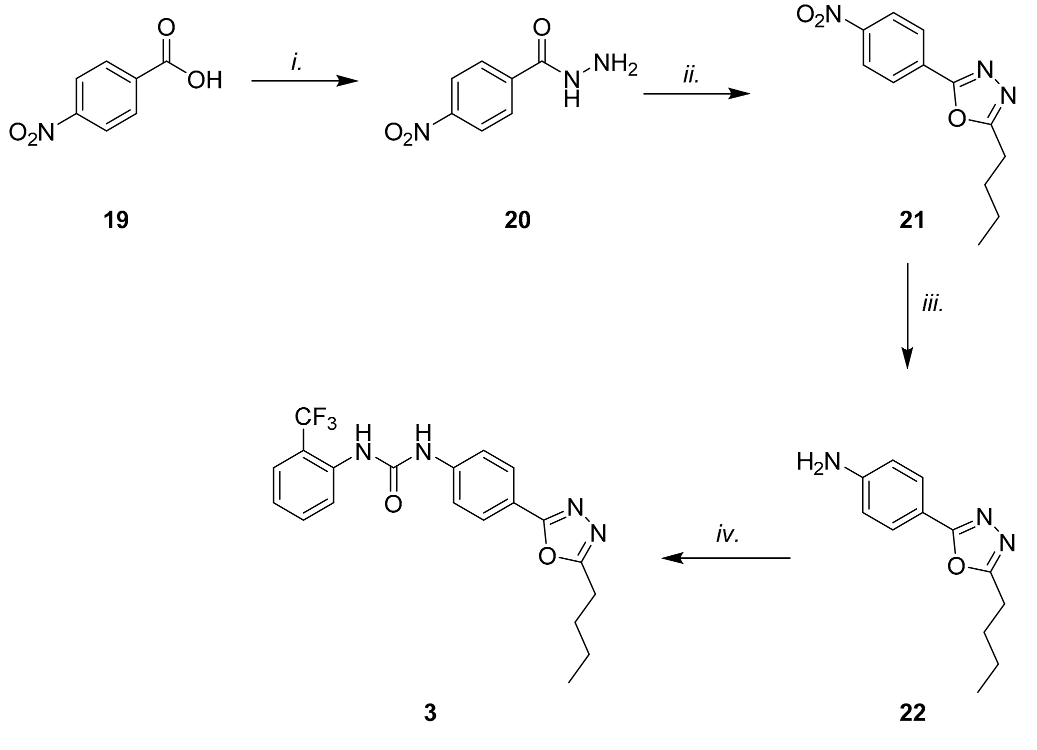

a. Reagents and conditions: *i. a)* H_2SO_4/EtOH, 100 °C, 3 h (Y=90%); *b)* $N_2H_4{\cdot}H_2O$, EtOH, 100 °C, 48 h (Y=85%); *ii. a)* Valeraldehyde, EtOH, 100 °C, 12 h; *b)* I_2, K_2CO_3, DMSO, 100 °C, 12 h (Y=70%); *iii.* H_2, Pd/C (10%), MeOH, 4 h (Y=99%); *iv.* 2-(trifluoromethyl)-phenylisocyanate, CH_2Cl_2 dry, rt., 12 h (Y=71%).

Ethyl 4-nitrobenzoate: 19 (200mg, 1.19 mmol), was solubilized in a mixture of H_2SO_4 (4mL) and EtOH (10mL). The mixture was stirred at 100 °C for 1h. After this time the solvent was partially evaporated at reduced pressure and the pH adjusted to 6 with $NaHCO_3$. The reaction was extracted with EtOAc, washed with Brine and dried over Na_2SO_4. Yield:90% ^{1}HNMR (400 MHz, $CDCl_3$): δ 8.26-8.24 (d, *J*= 8.8, 2H), 8.19-8.17 (d, *J*= 8.8Hz, 2H), 4.43-4.38 (q, *J*= 6.6 Hz, 2H), 1.42-1.38 (t, *J*= 6.9 Hz, 3H) ppm.

4-Nitrobenzohydrazide (20): To a solution of ethyl 4-nitrobenzoate (180 mg, 0.92 mmol) in EtOH (20mL), $N_2H_4{\cdot}H_2O$ (236 µL) was added. The resulting solution was heated to reflux for 48h. After this time the mixture was warmed at rt, and the volatiles were removed in vacuo. The residue was crystallized in ACN. Yield 85% ^{1}HNMR (400 MHz, MeOD-*d4*): δ 10.08 (bs, 1H), 8.28-8.26 (d, *J*= 8.8 Hz, 2H), 8.03-8.01 (d, *J*= 8.8Hz, 2H), 4.61 (bs, 2H) ppm.

2-Butyl-5-(4-nitrophenyl)-1,3,4-oxadiazole (21): A solution of valeraldehyde (58.7 µL, 0.55 mmol) and **20** (100 mg, 0.55 mmol) in EtOH, was heated at reflux for 12h. After this time, the solvent was removed at reduced pressure. The resulting residue was redissolved in DMSO (3mL) and K_2CO_3 (228.04 mg, 1.65 mmol) and I_2 (155.63 mg, 0.66 mmol) were added. The reaction mixture was stirred at 100°C for 12h. After completation of the reaction the mixture was cooled and treated with $Na_2S_2O_3$, then extracted with EtOAc (3x25mL), washed with Brine and dried over Na_2SO_4. The residue purified by flash chromatography. (PE/EtOAc 9:1). Yield:70%:^{1}HNMR (400 MHz, $CDCl_3$): δ 8.36-8.34 (d, *J*= 8.4 Hz, 2H), 8.22-8.20 (d, *J*= 8.4 Hz, 2H), 2.97-2.93 (t, *J*= 7.6 Hz, 2H), 1.86-1.82 (m 2H), 1.49-1.44 (m, 2H), 0.99-0.95 (t, *J*= 6.8 Hz, 3H) ppm.

4-(5-Butyl-1,3,4-oxadiazol-2-yl)aniline (22): Compound **21** (0.40 mmol) was solubilized in 30 mL of MeOH, and 10% Palladium on charcoal (5 mg) was added. The reaction mixture was stirred under Hydrogen atmosphere for 1h, then the mixture was filtered off on a celite pad. The solvent was evaporated at reduced pressure. Yield 99% Purification eluent. DCM/MeOH 98:2, ^{1}HNMR (400 MHz, $CDCl_3$): δ 7.80-7.78 (d, *J*= 8.4 Hz, 2H), 6.71-6.69 (d, *J*= 8.8 Hz, 2H), 4.07 (bs, 2H), 2.88-2.84 (t, *J*= 7.6 Hz, 2H), 1.82-1.75 (m, 2H), 1.48-1.39 (m, 2H), 0.96-0.93 (t, *J*= 7.6 Hz, 3H) ppm.

1-(4-(5-Butyl-1,3,4-oxadiazol-2-yl)phenyl)-3-(2-(trifluoromethyl)phenyl)urea (**3**): Aniline **22** (31 mg, 0.14 mmol) was added to a solution of 2-(trifluoromethyl)phenyl isocyanate (22 µL, 0.15 mmol) in anhydrous CH_2Cl_2 (15 mL) in one portion. The solution was stirred for 9 hours at r.t. under a nitrogen atmosphere. The solvent was removed at reduced pressure and the residue purified on silica to furnish the final product as white solid. (Purification eluent:PE/EtOAc 7:3).Yield 71%, white solid. ^{1}HNMR (400 MHz, MeOD-*d4*): δ 7.95-7.93 (m, 3H), 7.67-7.65 (m, 3H), 7.63-7.59 (t, *J*= 7. Hz, 1H), 7.31-7.27 (t, *J*= 7. Hz, 1H), 2.96-2.92 (t, *J*= 7.6, 2H), 1.86-1.79 (m, 2H), 1.51-1.42 (m, 2H), 1.01-0.97 (t, *J*= 7.2, 3H) ppm. ^{13}C-NMR (100 MHz, ACETONE-*d6*): δ 166.35, 164.11, 152.03, 142.81, 136.48, 132.79, 127.35, 125.90, 125.55, 124.60, 123.65, 118.51, 29.30, 28.92, 21.82, 12.95 ppm. HRMS (ESI) m/z calcd for $C_{20}H_{19}F_3N_4O_2$ $[M - H]^-$ 403.1382, found 403.1401. HPLC Purity: 97.5%.

Scheme S3. Synthesis of triazole 4[a]

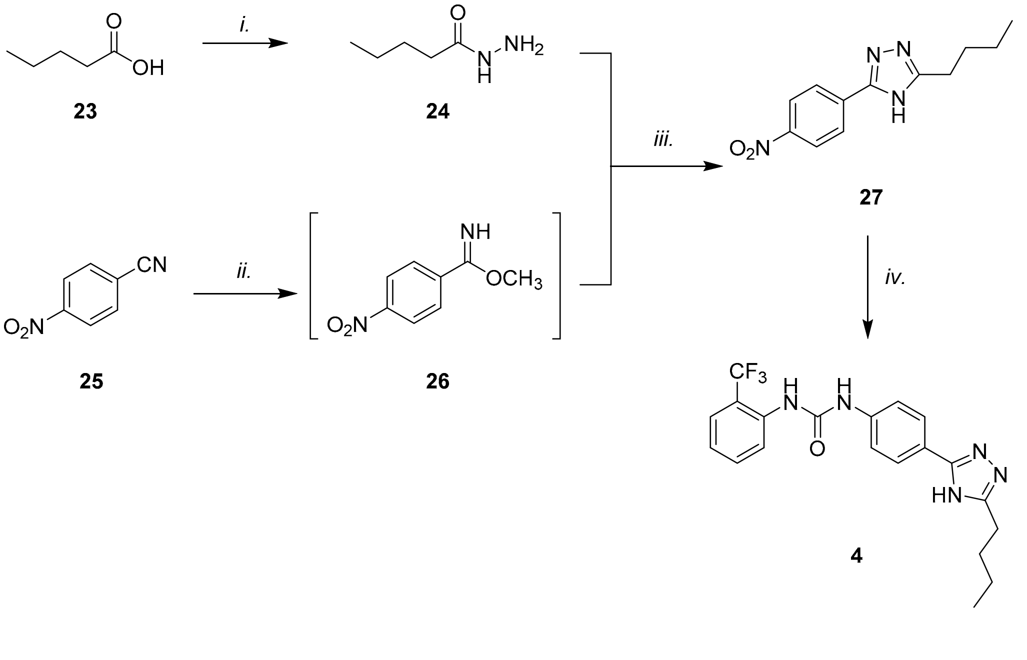

a. Reagents and conditions: *i. a)* EtOH/H_2SO_4, 100°C, 3 h (Y=99%)*; b)* $N_2H_4{\cdot}H_2O$, EtOH, 100 °C, 12 h. (Y=99%); *ii.* MeONa, dry MeOH, rt., 1h; *iii.* CH_3COOH, 0° C, 2 h (Y=60%); i*v. a)* H_2, Pd/C (10%), MeOH, 3h (Y=70%)*; b)* 2-(trifluoromethyl)-phenylisocyanate, CH_2Cl_2 dry, rt., 12h (Y=60%).

Ethyl pentanoate: 23 (1000mg, 9.79 mmol), was solubilized in a mixture of H_2SO_4 (4mL) and EtOH (10mL). The mixture was stirred at 100 °C for 3h. After this time the solvent was partially evaporated at reduced pressure and the pH adjusted to 6 with $NaHCO_3$. The reaction was extracted with EtOAc, washed with Brine and dried over Na_2SO_4. Yield 99%. ^{1}HNMR (400 MHz, $CDCl_3$): δ 4.14-4.08 (q, *J*= 7.2, 2H), 2.30-2.26 (t, *J*= 8.0 Hz, 2H), 1.62-1.56 (m,2H), 1.35-1.30 (t, *J*= 8.0 Hz, 3H), 1.28-1.24 (t, *J*= 7.9 Hz, 3H) ppm.

Pentanehydrazide (24): To a solution of ethyl pentanoate (420 mg, 3.22 mmol) in EtOH (20mL), $N_2H_4{\cdot}H_2O$ (783 μL) was added. The resulting solution was heated to reflux for 12h. After this time the mixture was warmed at rt, and the volatiles were removed in vacuo. **24** was used for further reactions without additional purification. Yield: 99% ^{1}HNMR (400 MHz, $CDCl_3$): δ 6.85 (bs, 1H), 3.87 (bs, 1H), 2.16-2.12 (t, *J*=7.6, 2H), 1.64-1.57 (m, 2H), 1.37-1.28 (m, 2H), 0.92-0.88 (t, *J*=7,2, 3H) ppm.

3-Butyl-5-(4-nitrophenyl)-4H-1,2,4-triazole (27): A 30% solution of MeONa (2.36 mmol) in anhydrous Methanol, was added dropwise to a solution of **25** (3.98 mmol) in CH_3OH. The reaction mixture was stirred at rt for 1h. The pH was adjusted to 6 with CH_3COOH at 0°C, then **24** (4.3 mmol) was added. The reaction was stirred at rt for 2h, then the solvent was removed at reduced pressure. Toluene (10 mL) was added and the reaction was heated at reflux with a Dean-Stark trap for 12 h. After this time the reaction was cooled and water and EtOAc were added. The mixture was stirred at rt for 30 min, then extracted, washed with Brine and dried over Na_2SO_4. The solvent was removed at reduced pressure and the residue was purified by flash chromatography. (PE/EtOAc 6:4). Yield 60%, ^{1}HNMR (400 MHz, MeOD-*d4)*: δ 8.37-8.24 (m, 4H), 7.89-7.87 (d, *J*= 7.6, 2H), 2.89-2.85(t, *J*=7.6 Hz, 2H), 1.84-1-76 (m, 2H), 1.46-1.39 (m, 2H), 0.98-0.94 (t, *J*=7.2 Hz, ,3H) ppm.^{13}C-NMR (100 MHz, ACETONE-*d6*): δ 160.05, 159.25, 148.07, 136.95, 127.13, 124.00, 29.98, 26.36, 22.29, 13.58 ppm.

4-(5-Butyl-4H-1,2,4-triazol-3-yl)aniline was synthesized following the synthetic procedure described for compound **22**. The residue was purified by flash chromatography (PE/EtOAc/TEA 6:4:0.5). Yield 70% ^{1}HNMR (400 MHz, $CDCl_3$): δ 7.78-7.76 (d, *J*=6.8 Hz, 2H), 6.68-6.66 (d, *J*=6.8 Hz, 2H), 4.13 (bs, 2H), 2.78-2.74 (t, *J*=7.6 Hz, 2H), 1.73-1.70 (m, 2H), 1.38-1.34 (m, 2H), 0.92-0.88 (t, *J*=7.2 Hz, 3H) ppm.

1-(4-(5-Butyl-4H-1,2,4-triazol-3-yl)phenyl)-3-(2-(trifluoromethyl)phenyl)urea (4): 4-(5-butyl-4H-1,2,4-triazol-3-yl)aniline (25 mg, 0.11 mmol) was added to a solution of 2-(trifluoromethyl)phenyl isocyanate (18 μL, 0.11 mmol) in anhydrous CH_2Cl_2 (15 mL) in one portion. The solution was stirred for 12 hours at r.t. under a nitrogen atmosphere. The solvent was removed at reduced pressure and the residue purified on silica to furnish the final product as white solid. (Purification eluent:PE/EtOAc 7:3).Yield 60%, white solid. ^{1}HNMR (400 MHz, MeOD-*d4*): δ 7.95-7.89 (m, 3H), 7.66-7.56 (m, 4H), 7.29-7.25 (t, *J*=7.6 Hz, 1H), 2.81-2.77 (t, *J*=7.6 Hz, 2H), 1.79-1.72 (m, 2H), 1.45-1.36 (m, 2H), 0.98-0.94 (t, *J*=7.6 Hz, 3H)ppm.^{13}C-NMR (100 MHz, MeOD-*d4*): δ 153.58, 146.64, 140.81, 135.86, 132.40, 126.77, 126.01, 125.67, 125.61, 124.01, 122.56, 121.78, 118.53, 29.31, 25.76, 21.88, 12.61 ppm. HRMS (ESI) m/z calcd for $C_{20}H_{20}F_3N_5O$ $[M - H]^-$ 403.1542, found 403.1580. HPLC Purity: 97.2%.

Scheme S4. Synthesis of tetrazoles 5-7[a]

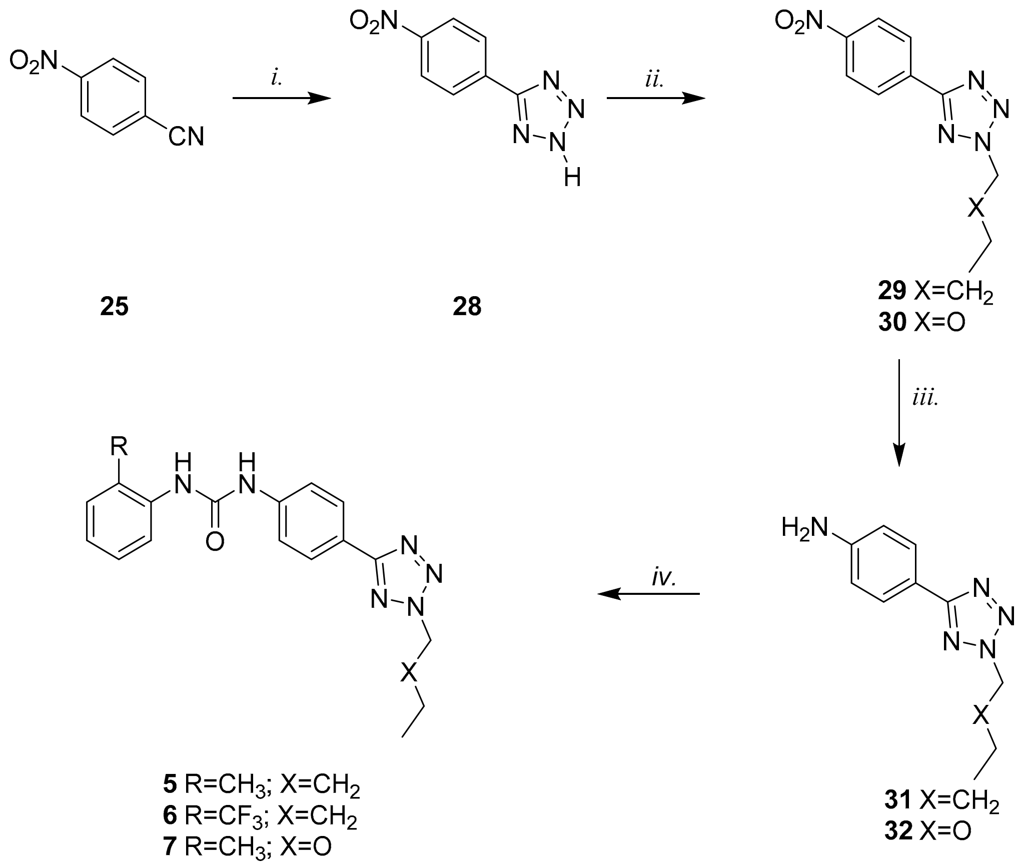

a. Reagents and conditions: *i.* NaN_3, NH_4Cl, DMF, 12 h, reflux (Y=80%); *ii.* K_2CO_3, 1-iodobutane or chloromethyl ethyl ether, CH_3CN, 12 h,. r.t (from 60 to 65%); *iii.* H_2, Pd/C, MeOH 30 min. (Y=99%); *iv.* Opportune isocyanate CH_2Cl_2, 9h r.t (from 62 to 73%).

5-(4-Nitrophenyl)-2H-tetrazole (28). A mixture of 4-nitrobenzonitrile (600 mg, 4.05 mmol) sodium azide (790 mg, 12.15 mmol) ammonium chloride (867 mg, 16.20 mmol) and DMF (5 mL) was heated at 120 °C for 12hr. Then the reaction was allowed to cool to r.t., water was added with continuous stirring. The mixture was then acidified to pH 2 with HCl 6N. The reaction mixture was extracted with EtOAc (3x20mL) and dried over Na_2SO_4, and the solvent was removed under reduced pressure, to give a yellow residue that was crystallized from Ethanol Yield 80% white solid ^{1}H N MR (MeOD-*d*$_4$): δ 8.40-8.38 (d, *J*= 7.2 Hz, 2H,), 8.28-8.28 (d, *J*= 8.0 Hz, 2H,) ppm. ^{13}C NMR (MeOD-*d*$_4$): δ 156.72, 149.46, 131.32, 128.18, 124.07 ppm. MS: m/z 189.9 [M-H]$^-$

2-Butyl-5-(4-nitrophenyl)-2H-tetrazole (29). A suspension of **28** (200 mg, 1.05 mmol), K_2CO_3 (174 mg, 1.26 mmol) and n-butyliodide (144μL, 1.26 mmol), in Acetonitrile was refluxed for 4 h. After that time, the reaction mixture was concentrated in vacuo, water was added and the residue was extracted with AcOEt (3x 25mL), washed with brine, and dried over Na_2SO_4. The resulting residue was purified by flash chromatography on silica gel (PE- DCM 1:8). Yield 65%, yellow solid. ^{1}H NMR (400 MHz $CDCl_3$): δ 8.26 (m, 4H), 4.67-4.63(t, *J*= 7.6Hz, 2H), 2.03-1.99 (m, 2H), 1.40-1.34 (m, 2H) 0.96-0.92 (t, *J*=7.2 Hz, 3H) ppm. ^{13}C NMR (100 MHz $CDCl_3$): δ 163.05, 148.74,133.42,127.53, 124.10, 53.20, 31.22, 19.57,13.30 ppm. MS: m/z 220 [M+H]$^+$

2-(Ethoxymethyl)-5-(4-nitrophenyl)-2H-tetrazole (30). A suspension of **28** (50 mg, 0.26 mmol), K_2CO_3 (43 mg, 0.31 mmol) and chloromethylethylether (28μL, 0.31 mmol), in Acetonitrile was refluxed for 12 h. After that time, the reaction mixture was concentrated in vacuo, water was added and the residue was extracted with AcOEt (3x 25mL), washed with brine, and dried over Na_2SO_4. The resulting residue was purified by flash chromatography on silica gel (PE- DCM 1:7). Yield 60%, yellow solid. ^{1}H NMR (Acetone-*d*$_6$): δ 8.41-8.38 (m, 4H), 6.08 (s, 2H), 3.74-3.71 (q, *J*= 6.2 Hz, 2H), 1.19-1.16 (t, *J*= 6.0 Hz, 3H) ppm; ^{13}C NMR (Acetone-*d*$_6$): δ 163.80, 149.22, 133.18, 127.83, 123.94, 81.90, 66.35, 14.34 ppm. MS: m/z 248.9 [M+H]$^+$

4-(2-Butyl-2H-tetrazol-5-yl)aniline (31). Aniline **31** was synthesized following the synthetic procedure described for compound **22**. Yield 99% ^{1}H NMR (400 MHz $CDCl_3$): δ 7.90-7.88 (d, *J*= 8.0Hz, 2H), 6.71-6.69 (d, *J*= 8.0Hz, 2H), 4.57-4.53 (t, *J*= 7.6Hz, 2H), 4.03 (s, 2H), 2.00-1.92, (m, 2H), 1.38-1.23 (m, 2H), 0.93-0.89 (t, *J*= 7.4 Hz, 3H) ppm. ^{13}C NMR (100 MHz, $CDCl_3$): δ 165.62, 148.77, 128.04, 117.56, 114.87, 52.69, 31.38, 19.56, 12.17 ppm MS (ESI): m/z 218.0 $[M+H]^+$, 239.9 $[M+Na]^+$.

4-(2-(Ethoxymethyl)-2H-tetrazol-5-yl)aniline (32). Aniline **32** was synthesized following the synthetic procedure described for compound **22**. Yield 99%^{1}H NMR (400 MHz $CDCl_3$): δ 7.97-7.95 (d, *J*= 7.2 Hz, 2H,), 6.75-6.73 (d, *J*= 7.2 Hz, 2H,), 5.870 (s, 2H), 3.88 (s, 2H), 3.70-3.68 (q, *J*= 6.7 Hz, 2H), 1.24-1.20 (t, *J*= 6.8 Hz, 3H), ppm. MS: m/z 220.2 $[M+H]^+$

1-(4-(2-Butyl-2H-tetrazol-5-yl)phenyl)-3-(o-tolyl)urea (5). Compound **31** (0.10 mmol) was added to a solution of o-tolyl isocyanate (0.15 mmol) in anhydrous MeOH (10 mL) in one portion. The solution was stirred for 9 hours at r.t. under a nitrogen atmosphere. The solvent was removed at reduced pressure and the residue purified on silica to furnish the final product as white solid. (DCM-MeOH 98:2). Yield 73% ^{1}H NMR (Acetone-*d*$_6$): δ 8.60 (s, 1H), 8.03-8.01 (d, *J*= 8.4Hz, 2H), 7.92-7.90 (d, *J*= 8.4 Hz, 1H), 7.71-7.69 (d, *J*= 8.4 Hz, 2H), 7.55 (s, 1H), 7.18-7.14 (m, 2H), 6.99-6.95 (t, *J*= 7.6Hz, 1H), 4.71-4.67 (t, *J*=, 2H), 2.27 (s, 3H), 2.03-1.98 (m, 2H), 1.43-1.34 (m, 2H), 0.97-0.94 (t, *J*= 7.4 Hz, 3H) ppm. ^{13}C NMR (Acetone-d6): δ 164.91, 152.54, 142.16, 137.33, 130.35, 128.48, 127.64, 126.40, 123.44, 122.20, 121.37, 118.75, 52.47, 31.09, 19.34, 17.17, 12.73 ppm. HRMS (ESI) m/z calcd for $C_{19}H_{22}N_6O$ $[M - H]^-$ 349.1777, found 349.1694. HPLC Purity: 99.6%.

1-(4-(2-Butyl-2H-tetrazol-5-yl)phenyl)-3-(2-(trifluoromethyl)phenyl)urea (6). Compound **31** (0.10 mmol) was added to a solution of o-trifluoromethyl-phenyl-isocyanate (0.15 mmol) in anhydrous MeOH (10 mL) in one portion. The solution was stirred for 9 hours at r.t. under a nitrogen atmosphere. The solvent was removed at reduced pressure and the residue purified on silica to furnish the final product as white solid. (DCM-MeOH 98:2). Yield 70%, white solid. ^{1}H NMR (400 MHz $CDCl_3$): δ 8.28 (s, 1H), 7.93-7.91 (d, *J*= 8.4 Hz,2H), 7.83-7.81 (d, *J*= 8.4 Hz, 1H), 7.47-7.34 (m, 3H), 7.09-7.05 (t, *J*= 7.2 Hz, 1H), 4.60-4.57 (t, *J*= 6.8 Hz, 2H), 2.01-1.97 (m, 2H), 1.39-1.33 (m, 2H), 0.95-0.91 (t, *J*= 7.2 Hz, 3H) ppm. ^{13}C NMR (100 MHz, $CDCl_3$): δ 164.70, 153.54, 140.20, 135.38, 132.54, 127.60, 126.29, 126.11, 125.23, 124.54, 122.43, 122.01, 120.07, 52.96, 31.27, 19.60, 13.34 ppm MS (ESI) m/z 405.1 $[M+H]^+$, 428.1 $[M+Na]^+$. HRMS (ESI) m/z calcd for $C_{19}H_{19}F_3N_6O$ $[M - H]^-$ 403.1494, found 403.1494. HPLC Purity: 99.5%.

1-(4-(2-(Ethoxymethyl)-2H-tetrazol-5-yl)phenyl)-3-(o-tolyl)urea (7). Compound **32** (0.10 mmol) was added to a solution of o-tolyl isocyanate (0.15 mmol) in anhydrous MeOH (10 mL) in one portion. The solution was stirred for 9 hours at r.t. under a nitrogen atmosphere. The solvent was removed at reduced pressure and the residue purified on silica to furnish the final product as white solid. (DCM-MeOH 98:2). Yield 62% ^{1}H NMR (400 MHz $CDCl_3$): δ 8.06-8.04 (d, *J*= 8.0 Hz, 2H,), 7.65-7.61 (m, 3H), 7.21-7.15 (m, 2H), 7.05-7.01 (t, *J*= 7.6 Hz, 1H,), 5.96 (s, 2H), 3.75-3.70 (q, *J*= 6.8 Hz, 2H), 2.30 (s, 3H), 1.21-1.17 (t, 3H, *J*= 6.8 Hz) ppm. ^{13}C NMR (100 MHz $CDCl_3$): δ 165.24, 142.06, 136.14, 130.47, 127.51, 126.33, 124.73, 123.05, 120.95, 118.61, 80.93, 66.18, 16.61, 13.32 ppm. HRMS (ESI) m/z calcd for $C_{18}H_{20}N_6O_2$ $[M - H]^-$ 351.1569, found 351.1591. HPLC Purity: 98.1%.

Scheme S5. Synthesis of thiazole 8[a]

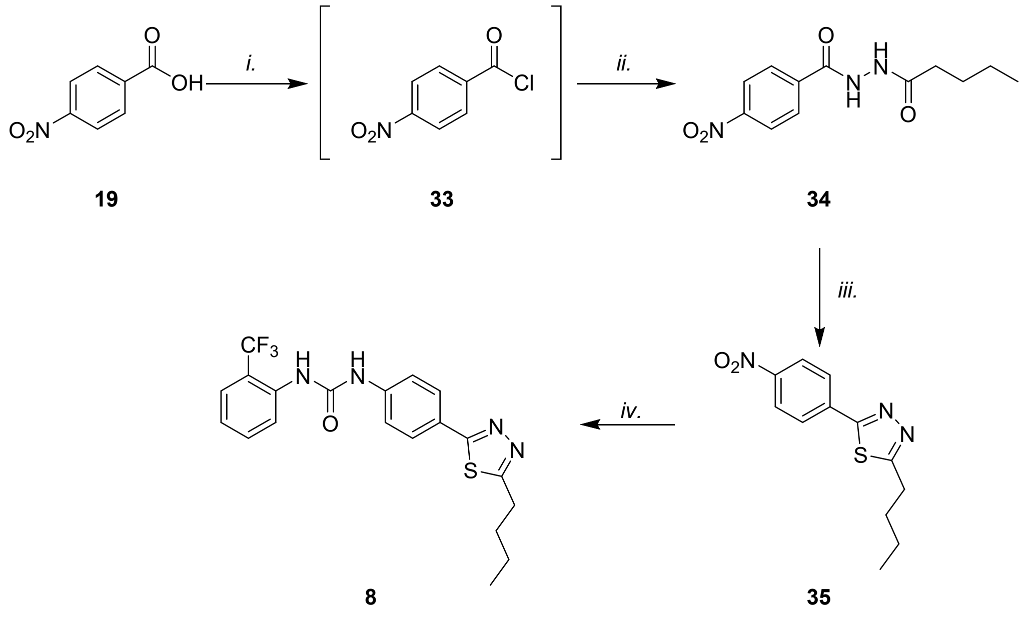

a. Reagents and conditions: *i.* $SOCl_2$, 100°C, 1 h; *ii.* **24**, DMAP, CH_2Cl_2, 0 °C, 12 h (Y=67%); *iii.* Lawesson's reagent, dioxane, 80 °C, 12h (Y=60%); *iv. a)* Fe^0, NH_4Cl, EtOH, H_2O, 80°C, 1h (Y=75%); *b)* 2-(trifluoromethyl)-phenylisocyanate, CH_2Cl_2 dry, rt., 12h (Y=68%).

4-Nitrobenzoyl chloride (33): 19 (719.8 mg, 4.30 mmol), was stirred with 1 mL of anhydrous $SOCl_2$, at 100°C, for 1 h. The excess of $SOCl_2$ was removed by distillation. **33** was used for further reactions without additional purification.

4-Nitro-N'-pentanoylbenzohydrazide (34): A solution of **33** (500 mg, 4.30 mmol) and DMAP (525 mg, 4.30 mmol) in dry DCM (5mL) was added dropwise to a solution of **24** in anhydrous DCM. The resulting mixture was stirred at rt on. The solvent was removed at reduced pressure and the residue purified by flash chromatography (DCM/MeOH/TEA 98:2:0.5), Yield: 67%, ^{1}HNMR (400 MHz, MeOD-d_4): δ 8.34-8.31 (d, *J*=8.8, 2H), 8.08-8.06 (d, *J*=8.8, 2H), 2.34-2.31 (t, *J*=7.2, 2H), 1.71-1.61 (m, 2H), 1.47-1.35 (m, 2H), 0.98-0.95 (t, *J*= 7.7 Hz, 3H) ppm.

2-Butyl-5-(4-nitrophenyl)-1,3,4-thiadiazole (35): Lawesson's reagent (458 mg, 1.31 mmol) was added to a stirring solution of **34** in anhydrous dioxane (20mL). The reaction mixture was stirred at 80°C for 24h. Dioxane was removed under reduced pressure, and the residue obtained was dissolved in water. The pH was basified to 9 by adding of $NaHCO_{3(aq)}$ and the organic phases were washed with brine and dried over Na_2SO_4. The residue was purified by flash chromatography (PE/EtOAc 8:2), Yield 60%^{1}HNMR (400 MHz, Acetone-d_6): δ 8.40-8.38 (d, *J*=8.8 Hz, 2H), 8.26-8.24 (d, *J*=8.8 Hz, 2H), 3.22-3.18 (t, *J*= 7.6 Hz, 2H), 1.87-1.80 (m, 2H), 1.52-1.42 (m, 2H), 0.98-0.94 (t, *J*=7.2 Hz, 3H) ppm. ^{13}C NMR (100 MHz, Acetone-d_6): δ 170.62, 168.98, 153.37, 141.94, 135.57, 132.57, 132.53, 128.66, 126.62, 126.10, 124.56, 124.14, 122.53, 119.50, 32.07, 29.84, 22.11, 13.60 ppm.

4-(5-Butyl-1,3,4-thiadiazol-2-yl)aniline: 35 (58 mg, 0.22 mmol) was solubilized in a mixture of EtOH (30mL) and water 2.5mL. To this Iron powder (62 mg, 1.1 mmol) and NH_4Cl (6 mg, 0.11 mmol) were added. The reaction mixture was heated at 80°C and stirred for 30 min. After this time the reaction was warmed to rt and filtered on a celite pad. The mixture was concentrated and water (15mL) was added, followed by extraction with EtOAc. The organic layers were washed with Brine and dried over Na_2SO_4. Yield 75%^{1}HNMR (400 MHz, $CDCl_3$): δ 7.73-7.71 (d, *J*=8.0, 2H), 6.71-6.69 (d, *J*=8.0, 2H), 3.82 (s, 2H), 3.08-3.06 (t, *J*=8.0, 2H), 1.79-1.77 (m, 2H), 1.46-1.44 (m, 2H), 0.96-0.93 (t, *J*=8.0 Hz, 3H) ppm. MS (ESI) m/z 234.1 $[M+H]^+$

1-(4-(5-Butyl-1,3,4-thiadiazol-2-yl)phenyl)-3-(2-(trifluoromethyl)phenyl)urea (8): 4-(5-butyl-1,3,4-thiadiazol-2-yl)aniline (25 mg, 0.11 mmol) was added to a solution of 2-(trifluoromethyl)phenyl isocyanate (17 µL, 0.11 mmol) in anhydrous CH_2Cl_2 (15 mL) in one portion. The solution was stirred for 12 hours at r.t. under a

nitrogen atmosphere. The solvent was removed at reduced pressure and the residue purified on silica to furnish the final product as white solid. (Purification eluent:PE/EtOAc 7:3).Yield 68%, white solid. ^{1}H NMR (400 MHz, $CDCl_3$) δ 8.93 (s, 1H), 7.89-7.85 (m, 2H), 7.74-7.72 (d, *J* = 7.9 Hz, 2H), 7.55-7.53 (d, *J* = 7.2Hz, 2H), 7.48-7.46 (d, *J* = 8.4 Hz, 2H), 7.17-7.14 (t, *J* = 7.5, 1H), 3.10-3.07 (t, *J* = 7.4Hz, 2H), 1.78-1.75 (m, 2H), 1.45-1.39 (m, 2H), 0.93-0.89 (t, *J* = 7.4 Hz, 3H) ppm. ^{13}C NMR (100 MHz $CDCl_3$):170.62, 168.98, 153.37, 141.94, 135.57, 132.53, 128.66, 126.62, 126.10, 124.56, 124.14, 122.52, 119.50, 32.07, 29.84, 22.11, 13.60 ppm. HRMS (ESI) m/z calcd for $C_{20}H_{19}F_3N_4OS$ $[M - H]^-$ 419.11, found 419.1121. HPLC Purity: 97.9%.

Scheme S6. Synthesis of triazoles 9 and 10[a]

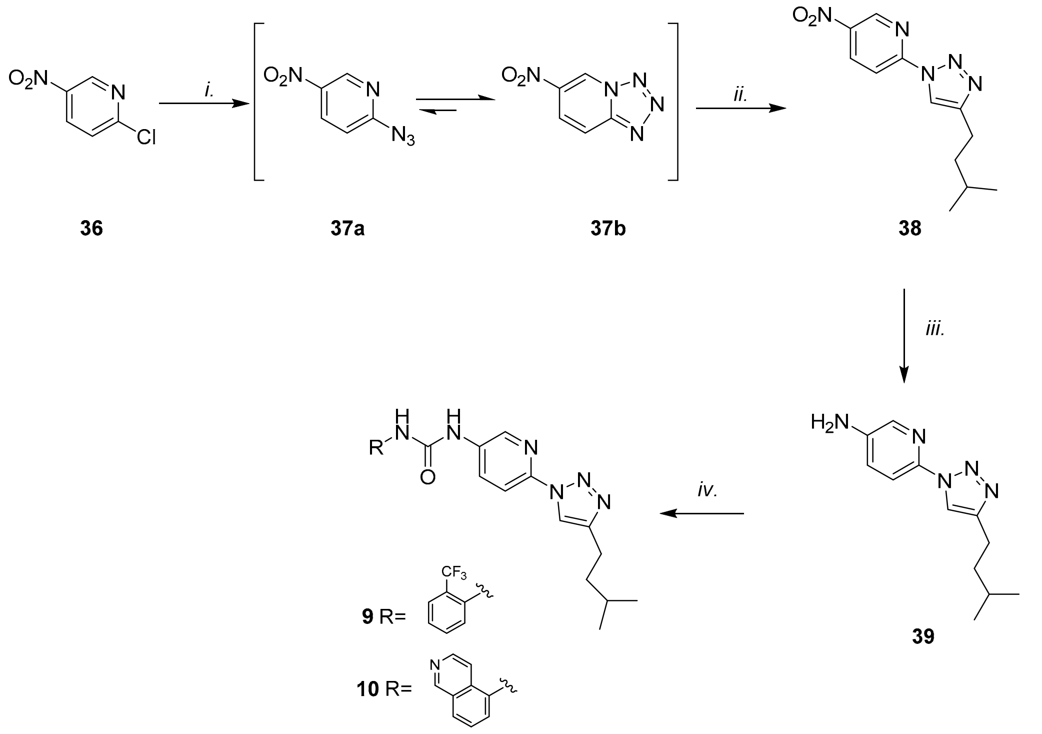

a. Reagents and conditions: *i.* NaN_3, HCl, EtOH: H_2O, reflux, 12 h; *ii.* $CuSO_4$·5 H_2O, sodium ascorbate, H_2O-THF (1:1), rt, 5h (Y=75% over two steps), *iii.* H_2, Pd/C, MeOH, 1h (Y=99%), *iv.* Opportune isocyanate, CH_2Cl_2, 5h, r.t (from 59 to 61%).

2-(4-Isopentyl-1H-1,2,3-triazol-1-yl)-5-nitropyridine (38). A solution of 2-chloro-5-nitropyridine **36** (100mg, 0.63 mmol) in a mixture of ethanol (8mL) and water (3mL) was carefully treated with NaN_3 (81mg, 1.26 mmol). Concentrated HCl (0.8 mL) was added dropwise at r.t. The reaction was stirred at reflux on, then cooled to rt. After that time saturated $NaHCO_3$ was added and the pH adjusted to 7. DCM (15 mL) was added and the rxn was washed with water. The organic layers were dried over Na_2SO_4 and concentrated to afford a yellow residue. The residue and the appropriate alkyne (90 µL, 0.75 mmol) were suspended in a 1:1 mixture of water and THF (1.5 mL each). To this, was added sodium ascorbate (1.0 equiv) and copper(II) sulfate pentahydrate (1.0 mmol). The mixture was stirred at r.t. for 5h. After that time the reaction was partitioned between sat. aq. solution of NH_4Cl and AcOEt, and stirred for 15 min. The organic layer was separated, dried over Na_2SO_4 and the solvent removed *in vacuo*. The residue was purified by flash chromatography on silica gel (DCM/MeOH 98:2). Yield 75%, yellow solid. ^{1}H NMR (400 MHz, $CDCl_3$): δ 9.28-9.28 (d, *J*=2.4 Hz, 1H),8.67-8.64 (dd, *J* = 8.8 Hz, *J* = 2.4 Hz, 1H), 8.36-8.32 (m, 2H), 2.80-2.76 (t, *J*=7.8 Hz, 2H), 1.67-1.58 (m, 3H), 0.84-0.82 (d, *J*=8.0Hz, 6H) ppm. ^{13}C NMR (100 MHz, $CDCl_3$): δ 152.24, 150.03, 144.99, 143.23, 134.59, 118.55, 114.44, 113.60, 38.08, 27.56, 23.48, 22.32 ppm. MS (ESI) m/z 260.3 $[M-H]^-$

6-(4-Isopentyl-1H-1,2,3-triazol-1-yl)pyridin-3-amine (39): Amine **39** was synthesized following the synthetic procedure described for compound **22**. The product was obtained as a pure compound. Yield 99%, yellow solid. Yield ^{1}H NMR (400 MHz, $CDCl_3$):δ 8.14 (s, 1H), 7.92-7.90 (m, 2H), 7.17-7.14 (dd, *J* = 8.8 Hz, *J* = 2.4 Hz, 1H), 3.89 (bs, 2H), 2.80-2.76 (t, *J*=7.8 Hz, 2H), 1.74-1.58 (m, 3H), 0.95-0.93 (d, *J*= 8.0 Hz, 6H) ppm. MS (ESI) m/z 232.3 $[M+H]^+$.

1-(6-(4-Isopentyl-1H-1,2,3-triazol-1-yl)pyridin-3-yl)-3-(2-(trifluoromethyl) phenyl)urea (9). Aniline **31** (0.10 mmol) was added to a solution of 2-(Trifluoromethyl)phenyl isocyanate (0.15 mmol) in anhydrous CH_2Cl_2 (15 mL) in one portion. The solution was stirred for 9 hours at r.t. under a nitrogen atmosphere. The solvent was

removed at reduced pressure and the residue purified on silica to furnish the final product as white solid. (Purification eluent: DCM-MeOH 98:2).Yield 61%, white solid. ^{1}H NMR (400 MHz, Acetone-d_6):δ 9.14 (s, 1H), 8.66 (s, 1H), 8.37 (s, 1H), 8.28-8.24 (d, *J*=8.0 Hz, 1H), 8.14-8.12 (d, *J*=8.0Hz, 1H), 8.06-8.04 (d, *J*=8.0 Hz, 1H), 7.81 (s, 1H), 7.70-7.64 (m, 2H), 7.33-7.30 (t, *J*=8.0 Hz, 1H), 2.80-2.75 (t, *J*=7.7 Hz, 2H), 1.64-1.61 (m, 3H), 0.96-0.94 (d, *J*=8.0 Hz, 6H) ppm. ^{13}C NMR (100 MHz, Acetone-d_6):δ 152.31, 148.41, 144.17, 138.61, 138.26, 136.46, 132.84, 128.80, 125.95, 125.65, 123.96, 117.98, 117.71, 113.35, 38.34, 27.33, 23.23, 21.91, 21.64 ppm. HRMS (ESI) m/z calcd for $C_{20}H_{21}N_6O$ [M - H]$^-$ 417.1615, found 417.1679. HPLC Purity: 99.3%.

1-(6-(4-Isopentyl-1H-1,2,3-triazol-1-yl)pyridin-3-yl)-3-(isoquinolin-5-yl)urea (10): (Purification eluent: DCM-MeOH 95:5).Yield 59%, white solid.^{1}H NMR (100 MHz, $CDCl_3$) δ = 9.05 (s, 1H), 8.98 (s, 1H), 8.31-8.30 (d, *J*=5.6 Hz,1H), 8.15-8.12 (m, 2H), 8.06-8.03 (d, *J*=12.0 Hz, 1H), 7.88-7.86 (d, *J*=8.8 Hz, 1H), 7.72-7.71 (d, *J*=4.4 Hz, 1H), 7.63-7.61 (d, *J*=8.0 Hz, 1H), 7.52-7.49 (t, *J*=7.8 Hz, 1H), 2.71-2.67 (t, *J*=7.4 Hz, 2H), 1.54-1.51 (m, 3H), 0.86-0.84 (d, *J*=6.0 Hz, 3H)ppm. ^{13}C NMR (100 MHz, $CDCl_3$) δ = 153.29, 152.43, 148.88, 143.67, 141.83, 138.35, 136.24, 132.67, 129.51, 129.01, 128.65, 127.75, 123.69, 123.14, 118.21, 114.56, 113.80, 38.18, 27.47, 23.34, 22.19. HRMS (ESI) m/z calcd for $C_{22}H_{23}N_7O$ [M - H]$^-$ 400.1886, found 400.1855. HPLC Purity: 99.5%.

Scheme S7. Synthesis of triazoles 11-14[a]

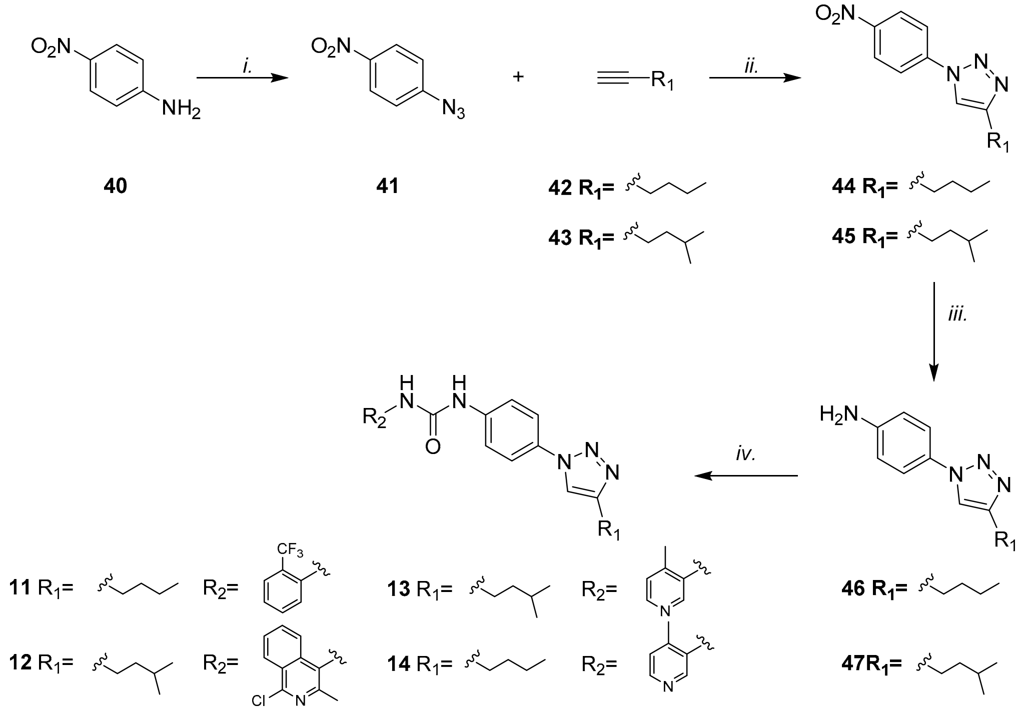

a. Reagents and conditions: *i.* a) *t*-BuONO, CH_3CN, 20 min. 0°C; b) $TMSiN_3$, CH_3CN, 2h r.t.; *ii.* $CuSO_4{\cdot}5\ H_2O$, sodium ascorbate, H_2O *t*BuOH (1:1), MW 120°C, 10 min; *iii.* H_2, Pd/C, MeOH, 1h, *iv.* Opportune isocyanate, CH_2Cl_2, 5h, r.t.

1-Azido-4-nitrobenzene (41)[24]. ^1H NMR(400 MHz, $CDCl_3$) δ 8.14-8.12 (d, *J*= 8.1 Hz, 2H), 7.07-7.05 (d, *J*= 8.1 Hz, 2H)ppm. MS (ESI) m/z 188.1 $[M+Na]^+$.

*General procedure for the preparation of compounds **44** and **45***

The appropriate alkyne **42** or **43** (2.17 mmol) and azide **41** (297 mg, 1.81 mmol) were suspended in 3 mL of a 1:1 mixture of water and *t*-BuOH in a 10 mL glass vial. Sodium ascorbate (1.81 mmol) and copper(II) sulfate pentahydrate (1.81mmol) were added and the mixture was then heated for 10 min. at 120°C under microwave irradiation, using an irradiation power of 300W. After this time the precipitate was filtered-off and purified on silica, to give the desired triazole compounds **44** or **45**.

4-Butyl-1-(4-nitrophenyl)-1H-1,2,3-triazole (44). (Purification eluent: DCM/MeOH 98:2). Yield 80%, yellow solid. Yield ^1H NMR (400 MHz, MeOD-*d4*): δ8.48 (s, 1H), 8.42-8.44 (d, *J*= 8.0 Hz, 2H), 8.12-8.10 (d, *J*= 8.0 Hz, 2H), 2.80-2.77 (t, *J*= 7.6 Hz, 2H), 1.74-1.78 (m, 2H), 1.46-1.40 (m, 2H), 0.99-0.952 (t, *J*= 7.2 Hz, 3H) ppm.MS (ESI) m/z 245 $[M-H]^-$, 281 $[M+Cl]^-$.

4-Isopentyl-1-(4-nitrophenyl)-1H-1,2,3-triazole (45). (Purification eluent: PE/EtOAc 9:1).Yield 86%, yellow solid. ^1H NMR (400 MHz, MeOD-*d4*):δ8.45-8.39 (m, 3H), 8.11-8.09 (d, *J*= 8.0 Hz, 2H), 2.81-2.77 (t, 7.6 Hz, 2H), 1.64-1.61 (m, 3H), 0.98-0.96 (d, *J*= 7.4 Hz, 2H) ppm. MS (ESI) m/z 260.9 $[M+H]^+$.

4-Butyl-1-(4-aminophenyl)-1H-1,2,3-triazole (46). Aniline **46** was synthesized following the synthetic procedure described for compound **22**. (Purification eluent: DCM/MeOH 95:5).Yield 80%, white solid. ^1H NMR (400 MHz, MeOD-*d4*):δ7.98 (s, 1H), 7.43-7.41 (d, *J*= 8.0 Hz, 2H), 6.78-6.76 (d, *J*= 8.0 Hz, 2H), 2.72-2.68 (t, *J*= 7.6 Hz, 2H), 1.70-1.64 (m, 2H), 1.40-1.35 (m, 2H), 0.95-0.90 (t, *J*= 7.1 Hz, 3H) ppm.MS (ESI) m/z 217 $[M+H]^+$, 240 $[M+Na]^+$.

4-Isopentyl-1-(4-aminophenyl)-1H-1,2,3-triazole (47). Aniline **47** was synthesized following the synthetic procedure described for compound **22**. (Purification eluent: DCM/MeOH 98:2).Yield 86%, yellow solid. Yield ^1H NMR (400 MHz, $CDCl_3$):δ7.99 (s, 1H), 7.43-7.41 (d, *J*= 7.8 Hz, 2H), 6.78-6.76 (d, *J*= 7.8Hz, 2H), 4.84 (s, 2H), 2.74-2.70 (t, *J*=7.6 Hz, 2H), 1.59-1.56 (m, 3H), 0.94-0.92 (d, *J*= 7.4 Hz, 6 H) ppm.MS (ESI) m/z 245 $[M-H]^-$, 281 $[M+Cl]^-$.

*General procedure for the preparation of compounds **11-14***

The opportune aniline **46** or **47** (100 mg, 0.46 mmol) was added to a solution of the appropriate isocyanate (85 μL, 0.65 mmol) in anhydrous CH_2Cl_2 (10 mL) in one portion. The solution was stirred for 4 hours at r.t. under a nitrogen atmosphere. The solvent was removed, at reduced pressure and the residue purified by flash chromatography using the opportune eluent.

1-(4-(4-Butyl-1H-1,2,3-triazol-1-yl)phenyl)-3-(2-(trifluoromethyl)phenyl)urea (11). (Purification eluent: DCM/MeOH 98:2).Yield 78%, white solid. ^{1}HNMR (400 MHz, MeOD-*d4*): δ 8.16 (s, 1H), 7.93-7.92 (d, *J*=8.0 Hz, 2H), 7.71-7.68 (m, 2H), 7.64-7.61 (m, 3H), 7.59-7.55 (t, *J*=7.8 Hz, 1H), 7.27-7.23 (t, *J*= 8.0 Hz, 1H), 2.75-2.71 (t, *J*=7.6 Hz, 2H), 1.72-1.64 (m, 2H), 1.42-1.34 (m, 2H), 0.96-0.92 (t, *J*= 7.6 Hz, 3H) ppm ^{13}C NMR (100 MHz, MeOD-*d4*): δ 153.59, 148.64, 139.83, 136.14, 132.43, 131.98, 126.07, 125.71, 124.12, 120.79, 119.82, 119.33, 31.28, 24.61, 21.88, 12.71 ppm. HRMS (ESI) m/z calcd for $C_{20}H_{20}F_3N_5O$ $[M - H]^-$ 402.1542, found 402.1599. HPLC Purity: 99.6%.

1-(1-Chloro-3-methylisoquinolin-4-yl)-3-(4-(4-isopentyl-1H-1,2,3-triazol-1-yl)phenyl)urea (12): (Purification eluent: DCM/MeOH 98:2).Yield 60%, white solid. ^{1}HNMR (400 MHz, MeOD-*d4*): δ 8.34-8.32 (d, *J*=8.4 Hz, 1H), 8.21 (s, 1H), 8.08-8.06 (d, *J*=8.4 Hz, 1H), 7.89-7.85 (t, *J*=7.6 Hz, 1H), 7.74, 7.20 (m, 3H), 7.67-7.65 (d, *J*=8.4 Hz, 2H), 2.79-2.75 (t, *J*= 7.2 Hz, 2H), 2.62 (s, 3H), 1.64-1.61 (m, 3H), 0.93-0.92 (d, *J*=6.0 Hz, 6H) ppm. ^{13}CNMR (100 MHz $CDCl_3$): δ = 153.28, 153.18, 151.88, 147.67, 136.75, 135.75, 133.97, 129.85, 128.14, 127.77, 127.55, 125.89, 123.06, 123.03, 121.02, 119.62, 38.27, 27.88, 27.45, 22.84, 21.44 ppm. HRMS (ESI) m/z calcd for $C_{24}H_{25}ClN_6O$ $[M - H]^-$ 447.1700, found 447.1658. HPLC Purity: 97.2%.

1-(4-(4-Isopentyl-1H-1,2,3-triazol-1-yl)phenyl)-3-(4-methylpyridin-3-yl)urea (13). The residue was purified by flash chromatography on silica gel (DCM/MeOH 98:2).Yield 74%, white solid.^{1}H NMR (400 MHz, MeOD-*d4*):δ8.67 (s, 1H), 8.20 (s, 1H), 8.15-8.14 (d, *J*= 4.8 Hz, 1H), 7.84-7.81 (d, *J*= 8.0 Hz, 2H), 7.67-7.65 (*J*= 8.0 Hz, 2H), 7.30-7.29 (d, *J*=4.8 Hz, 1H), 2.78-2.74 (t, *J*= 8.0 Hz, 2H), 2.35 (s, 3H), 1.63-1.60 (m, 3H), 0.97-0.95 (d, *J*= 8.0 Hz, 6H) ppm. ^{13}C-NMR (100 MHz, MeOD-*d4*):δ164.69, 153.67, 143.82, 143.19, 141.39, 140.12, 134.50, 127.09, 125.50, 121.37, 118.72, 38.28, 27.38, 22.87, 21.32, 16.59 ppm. HRMS (ESI) m/z calcd for $C_{19}H_{22}N_6O$ $[M - H]^-$ 349.1777, found 349.1777. HPLC Purity: 98.3%.

1-(4-(4-Butyl-1H-1,2,3-triazol-1-yl)phenyl)-3-(4-methylpyridin-3-yl)urea (14). The residue was purified by flash chromatography on silica gel (DCM/MeOH 98:2).Yield 68%, white solid.^{1}H NMR (400 MHz, MeOD-*d4*):δ8.87 (s, 1H), 8.20 (s, 1H), 8.15-8.14 (d, *J*= 4Hz, 1H), 7.74-7.71 (d, *J*= 8.0 Hz, 2H), 7.65-7.63 (*J*= 8.0 Hz, 2H), 7.30-7.29 (d, *J*=4Hz, 1H), 4.70-4.67 (t, *J*= 7.0 Hz, 2H), 2.36 (s,3H), 2.06-1.99 (m, 2H), 1.41-1.36 (m, 2H), 1.00-0.96 (t, *J*= 8.0 Hz, 3H) ppm. ^{13}C-NMR (100 MHz, MeOD-*d4*): δ164.69, 153.67, 143.82, 143.19, 141.39, 140.12, 134.50, 127.09, 125.50, 121.37, 118.72, 50.61, 30.06, 18.51, 14.20, 11.30 ppm. HRMS (ESI) m/z calcd for $C_{20}H_{24}N_6O$ $[M - H]^-$ 363.1933, found 363.1976. HPLC Purity: 98.1%.

In vitro ADME assays:

Chemicals. All solvents, reagents, were from Sigma-Aldrich Srl (Milan, Italy). Dodecane was purchased from Fluka (Milan, Italy). Pooled Male Donors 20 mg mL^{-1} HLM were from BD Gentest-Biosciences (San Jose, California). Milli-Q quality water (Millipore, Milford, MA, USA) was used. Hydrophobic filter plates (MultiScreen-IP, Clear Plates, 0.45 μm diameter pore size), 96-well microplates, and 96-well UV-transparent microplates were obtained from Millipore (Bedford, MA, USA).

UV/LC-MS method

For the quantitative analysis was used an UV/LC-MS system. LC analysis were performed by Agilent 1100 LC/MSD VL system (G1946C) (Agilent Technologies, Palo Alto, CA) constituted by a vacuum solvent degassing unit, a binary high-pressure gradient pump, an 1100 series UV detector and a 1100 MSD model VL benchtop mass spectrometer was used. The Agilent 1100 series mass spectra detection (MSD) single-quadrupole instrument was equipped with the orthogonal spray API-ES (Agilent Technologies, Palo Alto, CA). Nitrogen was used as nebulizing and drying gas. The pressure of the nebulizing gas, the flow of the drying gas, the capillary voltage, the fragmentor voltage and the vaporization temperature were set at 40 psi, 9 L min-1, 3000 V, 70 V and 350°C, respectively. UV detection was monitored at 280 nm. The LC-ESI-MS determination was performed by operating the MSD in the positive ion mode. Spectra were acquired over the scan range m/z 50-1500 using a step size of 0.1 u. Chromatographic analysis was performed using a Varian Polaris 5 C18-A column (150 x 4.6 mm, 5 µm particle size) at room temperature. Analysis was carried out using gradient elution of a binary solution; eluent A was ACN, while eluent B consisting of water. The analysis started at 0% A for three minutes, then rapidly increased up to 98% in 12 min and finally remaining at 98% A until 18 min. The analysis was performed at flow rate of 0.8 mL min-1 and injection volume was 20 µL.

Parallel Artificial Membrane Permeability Assay (PAMPA):

Donor solution (0.5 mM) was prepared by diluting 1 mM dimethylsulfoxide (DMSO) compound stock solution using phosphate buffer (pH 7.4, 0.025 M). Filters were coated with 5 μL of a 1% (w/v) dodecane solution of phosphatidylcholine prepared from $CHCl_3$ solution 10% w/v, for intestinal permeability. Donor solution (150 μL) was added to each well of the filter plate. To each well of the acceptor plate were added 300 μL of solution (50% DMSO in phosphate buffer). All compounds were tested in three different plates on different days. The sandwich was incubated for 5 h at room temperature under gentle shaking. After the incubation time, the plates were separated, and samples were taken from both receiver and donor sides and analyzed using LC with UV detection at 280 nm.

LC analysis were performed with a Varian Prostar HPLC system (Varian Analytical Instruments, USA) equipped with a binary pump with a manual injection valve and model Prostar 325 UV-VIS Detector. Chromatographic separation were conducted using a Polaris C18-A column (150-4.6 mm, 5 μm particle size) at a flow rate of 0.8 mL min^{-1} with a mobile phase composed of 60% ACN/40% H_2O.

Permeability (Papp) for PAMPA was calculated according to the following equation, obtained from Wohnsland and Faller and Sugano *et al*. equation with some modification in order to obtain permeability values in cm s^{-1},

$$P_{app} = \frac{V_D V_A}{(V_D + V_A) A t} - \ln(1 - r)$$

where V_A is the volume in the acceptor well, V_D is the volume in the donor well (cm^3), A is the “effective area” of the membrane (cm^2), t is the incubation time (s) and r the ratio between drug concentration in the acceptor and equilibrium concentration of the drug in the total volume (V_D+V_A). Drug concentration is estimated by using the peak area integration.

Membrane retentions (%) were calculated according to the following equation:

$$\%MR = \frac{[r-(D+A)]100}{Eq}$$

where *r* is the ratio between drug concentration in the acceptor and equilibrium concentration, *D, A,* and *Eq* represented drug concentration in the donor, acceptor and equilibrium solution, respectively.

Water Solubility Assay.

Each solid compound (1 mg) was added to 1 mL of water. The samples were shaked in a shaker bath at room temperature for 24-36 h. The suspensions were filtered through a 0.45 μm nylon filter (Acrodisc), and the solubilized compound determined by LC-MS-MS assay. For each compound the determination was performed in triplicate.

For the quantification was used an LC-MS system consisted of a Varian apparatus (Varian Inc) including a vacuum solvent degassing unit, two pumps (212-LC), a Triple Quadrupole MSD (Mod. 320-LC) mass spectrometer with ES interface and Varian MS Workstation System Control Vers. 6.9 software. Chromatographic separation was obtained using a Pursuit C18 column (50 x 2.0 mm) (Varian) with 3 μm particle size and gradient elution: eluent A being ACN and eluent B consisting of water. The analysis started with 0% of eluent A, which was linearly increased up to 70% in 10 min, then slowly increased up to 98% up to 15 min. The flow rate was 0.2 mL min^{-1} and injection volume was 5 μL. The instrument operated in positive mode and parameters were: detector 1850 V, drying gas pressure 25.0 psi, desolvation temperature 300.0 °C, nebulizing gas 40.0 psi, needle 5000 V and shield 600 V. Nitrogen was used as nebulizer gas and drying gas. Collision induced dissociation was performed using Argon as the collision gas at a pressure of 1.8 mTorr in the collision cell.

Microsomal Stability Assay.

Each compound in DMSO solution was incubated at 37 °C for 60 min in 125 mM phosphate buffer (pH 7.4), 5 μL of human liver microsomal protein (0.2 mg mL^{-1}), in the presence of a NADPH-generating system at a final volume of 0.5 mL (compound final concentration, 50 μM); DMSO did not exceed 2% (final solution). The reaction was stopped by cooling on ice and adding 1.0 mL of acetonitrile. The reaction mixtures were then centrifuged, and the parent drug and metabolites were subsequently determined by LC-UV-MS.

Chromatographic analysis was performed with an Agilent 1100 LC/MSD VL system (G1946C) (Agilent Technologies, Palo Alto, CA) constituted by a vacuum solvent degassing unit, a binary high-pressure gradient pump, an 1100 series UV detector, and an 1100 MSD model VL benchtop mass spectrometer.

Chromatographic separation was obtained using a Varian Polaris C18-A column (150-4.6 mm, 5 μm particle size) and gradient elution: eluent A being ACN and eluent B consisting of water. The analysis started with 2% of eluent A, which was rapidly increased up to 70% in 12 min, then slowly increased up to 98% in 20 min. The flow rate was 0.8 mL min^{-1} and injection volume was 20 μL.

The Agilent 1100 series mass spectra detection (MSD) single-quadrupole instrument was equipped with the orthogonal spray API-ES (Agilent Technologies, Palo Alto, CA). Nitrogen was used as nebulizing and drying gas. The pressure of the nebulizing gas, the flow of the drying gas, the capillary voltage, the fragmentor voltage, and the vaporization temperature were set at 40 psi, 9 L/min, 3000 V, 70 V, and 350 °C, respectively. UV detection was monitored at 280 nm. The LC-ESI-MS determination was performed by operating the MSD in the positive ion mode. Spectra were acquired over the scan range *m/z* 100-1500 using a step size of 0.1 u. The percentage of not metabolized compound was calculated by comparison with reference solutions
